# Functional characterization of key enzymes involved in the biosynthesis of distinctive flavonoids and stilbenoids in *Morus notabilis*

**DOI:** 10.1093/hr/uhaf171

**Published:** 2025-07-07

**Authors:** Meng-Wen Hu, Jie Fu, Ying Lu, Xin-Yan Liu, Jiao-Zhen Zhang, Jiang-Nan Li, Dan-Dan Xu, Ya-Tong Li, Pei-Xi Hao, Ming-Xin Cui, Lin-Lin Zhi, Hong-Xiang Lou, Ai-Xia Cheng

**Affiliations:** State Key Laboratory of Discovery and Utilization of Functional Components in Traditional Chinese Medicine, Key Laboratory of Chemical Biology (Ministry of Education), School of Pharmaceutical Sciences, Cheeloo College of Medicine, Shandong University, Jinan, Shandong 250012, China; State Key Laboratory of Discovery and Utilization of Functional Components in Traditional Chinese Medicine, Key Laboratory of Chemical Biology (Ministry of Education), School of Pharmaceutical Sciences, Cheeloo College of Medicine, Shandong University, Jinan, Shandong 250012, China; State Key Laboratory of Discovery and Utilization of Functional Components in Traditional Chinese Medicine, Key Laboratory of Chemical Biology (Ministry of Education), School of Pharmaceutical Sciences, Cheeloo College of Medicine, Shandong University, Jinan, Shandong 250012, China; State Key Laboratory of Discovery and Utilization of Functional Components in Traditional Chinese Medicine, Key Laboratory of Chemical Biology (Ministry of Education), School of Pharmaceutical Sciences, Cheeloo College of Medicine, Shandong University, Jinan, Shandong 250012, China; State Key Laboratory of Discovery and Utilization of Functional Components in Traditional Chinese Medicine, Key Laboratory of Chemical Biology (Ministry of Education), School of Pharmaceutical Sciences, Cheeloo College of Medicine, Shandong University, Jinan, Shandong 250012, China; State Key Laboratory of Discovery and Utilization of Functional Components in Traditional Chinese Medicine, Key Laboratory of Chemical Biology (Ministry of Education), School of Pharmaceutical Sciences, Cheeloo College of Medicine, Shandong University, Jinan, Shandong 250012, China; State Key Laboratory of Discovery and Utilization of Functional Components in Traditional Chinese Medicine, Key Laboratory of Chemical Biology (Ministry of Education), School of Pharmaceutical Sciences, Cheeloo College of Medicine, Shandong University, Jinan, Shandong 250012, China; State Key Laboratory of Discovery and Utilization of Functional Components in Traditional Chinese Medicine, Key Laboratory of Chemical Biology (Ministry of Education), School of Pharmaceutical Sciences, Cheeloo College of Medicine, Shandong University, Jinan, Shandong 250012, China; State Key Laboratory of Discovery and Utilization of Functional Components in Traditional Chinese Medicine, Key Laboratory of Chemical Biology (Ministry of Education), School of Pharmaceutical Sciences, Cheeloo College of Medicine, Shandong University, Jinan, Shandong 250012, China; State Key Laboratory of Discovery and Utilization of Functional Components in Traditional Chinese Medicine, Key Laboratory of Chemical Biology (Ministry of Education), School of Pharmaceutical Sciences, Cheeloo College of Medicine, Shandong University, Jinan, Shandong 250012, China; State Key Laboratory of Discovery and Utilization of Functional Components in Traditional Chinese Medicine, Key Laboratory of Chemical Biology (Ministry of Education), School of Pharmaceutical Sciences, Cheeloo College of Medicine, Shandong University, Jinan, Shandong 250012, China; State Key Laboratory of Discovery and Utilization of Functional Components in Traditional Chinese Medicine, Key Laboratory of Chemical Biology (Ministry of Education), School of Pharmaceutical Sciences, Cheeloo College of Medicine, Shandong University, Jinan, Shandong 250012, China; State Key Laboratory of Discovery and Utilization of Functional Components in Traditional Chinese Medicine, Key Laboratory of Chemical Biology (Ministry of Education), School of Pharmaceutical Sciences, Cheeloo College of Medicine, Shandong University, Jinan, Shandong 250012, China

## Abstract

The mulberry (*Morus notabilis*) is a medicinal and edible plant and contains diverse flavonoids and stilbenoids with significant medicinal benefits. The biosynthesis of these compounds has only been partially elucidated. In the present investigation, we identified and characterized two 4-coumarate: coenzyme A ligases (Mn4CL1 and Mn4CL2), two polyketide synthases (MnCHS and MnSTS), three chalcone reductases (MnCHR1–3), and two 2-oxoglutarate-dependent dioxygenases (MnFLS and MnF3H) involved in flavonoids and stilbenoid biosynthesis. MnCHS converts *p*-coumaroyl-CoA into naringenin and facilitates the novel conversion of 2,4-dihydroxycinnamoyl-CoA to steppogenin, which features hydroxyl groups at the 4′ and 6′ positions on the B ring. MnSTS could convert *p*-coumaroyl-CoA and 2,4-dihydroxycinnamoyl-CoA into resveratrol and oxyresveratrol, respectively. Furthermore, MnCHR1 was first identified in mulberry and collaborated with MnCHS to produce isoliquiritigenin and 2,4,2′,4′-tetrahydroxychalcone. A co-expression system of Mn4CL1, MnCHS, and MnCHR1 enabled the fermentation production of steppogenin and 2,4,2′,4′-tetrahydroxychalcone in engineered *Escherichia coli*. The *in vitro* enzymatic assays confirmed that MnFLS showed both FLS and F3H activities, whereas transgenic experiments revealed its predominant FLS function *in vivo*; MnF3H was confirmed as a bona fide F3H. These findings provide new insights into the flavonoids and stilbenoids biosynthesis pathway in mulberry and suggest a potential parallel pathway for 4′,6′-dihydroxylated flavonoids biosynthesis.

## Introduction

**Figure 1:**
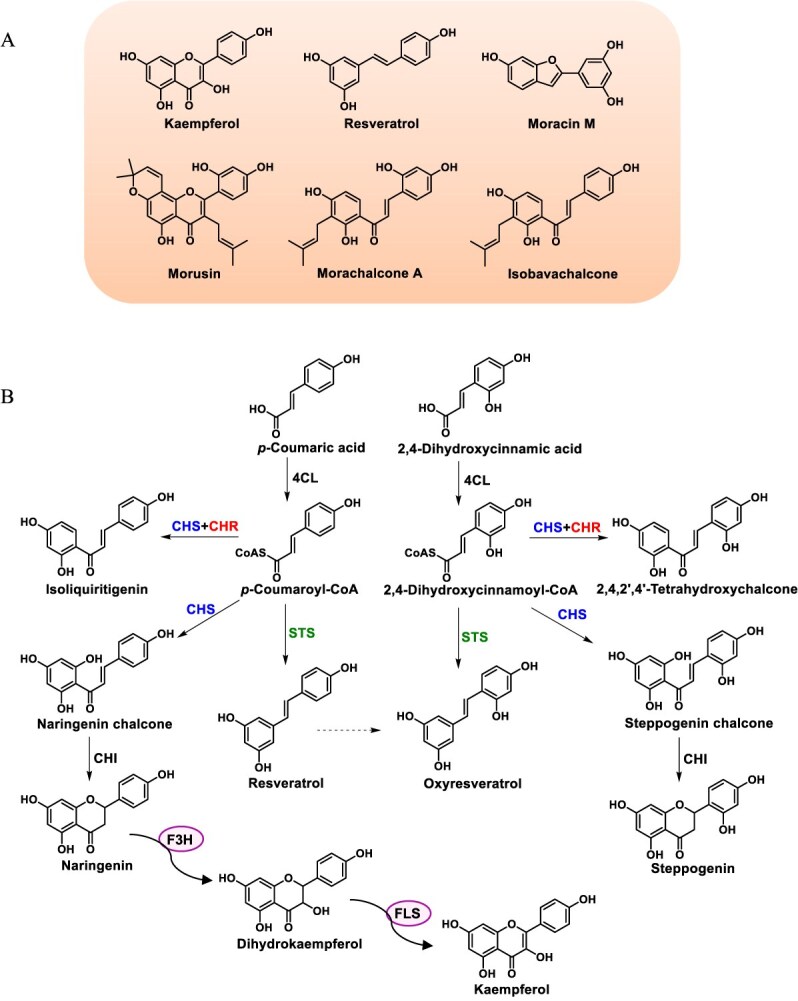
Representative flavonoids and stilbenoids in *M. notabilis*. (A) Structure of representative flavonoids and stilbenoids. (B) The proposed biosynthetic pathway of flavonoids and stilbenoids in *M. notabilis*.

*Morus notabilis* is a fast-growing deciduous plant within the Moraceae family and has been cultivated by humans for over 5000 years [1, 2]. It is recognized as a source of both medicinal and edible components and has consistently aroused attention for herbal and nutritional values. Mulberry was found to possess antidiabetic, neuroprotective, anti-oxidative, anti-inflammatory, anti-atherosclerotic, and anticancer properties [3, 4]. The plant contains plentiful flavonoids and stilbenoids, as well as complex derivatives [5] (Fig. 1A). Notably, in addition to the common flavonoids and stilbenoids, there are some distinctive flavonoids characterized by hydroxyl groups at the 4′ and 6′ positions of the B ring (4′,6′-dihydroxylated flavonoids) [6]. Diverse compounds endow mulberry with promising potential for drug development.

It is well known that flavonoids and stilbenoids are synthesized via the phenylpropanoid pathway in plants [7, 8]. The pivotal intermediate naringenin is synthesized through sequential catalytic reactions involving chalcone synthase (CHS) and chalcone isomerase (CHI). Subsequently, flavanone 3β-hydroxylase (F3H) performs the 3β-hydroxylation of naringenin yielding dihydroflavonol, which can be further converted into flavonol via flavonol synthase (FLS). This represents the routine flavonoid biosynthetic pathway in mulberry. Furthermore, mulberry is one of the specific plants capable of producing stilbenoids such as resveratrol, which is catalyzed by stilbene synthase (STS).

CHS initiates the first committed step in the flavonoid biosynthetic pathway. It is highly conserved in different plants [9] and is the most extensively studied type III plant polyketide synthase (PKS) [10]. The resolution of the X-ray crystal structure of *Medicago sativa* CHS showed that it is a homodimer of two relatively modest-sized proteins of 40–45 kDa, containing a conserved Cys–His–Asn catalytic triad in internal active sites [11]. The majority of reported CHS enzymes preferentially utilize *p*-coumaroyl-CoA as a substrate, but some individual CHSs, such as ScCHS1, exhibit a preference for feruloyl-CoA and caffeoyl-CoA [12]. STS, another enzyme within the type III PKS superfamily, plays a critical role in stilbene biosynthesis and shares high sequence homology with CHS [13]. Despite starting from the same substrates and synthesizing the same linear tetraketide intermediate, CHS and STS employ distinct condensation mechanisms ultimately generating different skeletal structures [14]. Specifically, STS cyclizes the resulting tetraketide intermediate via an intramolecular C2 → C7 aldol condensation, distinct from the intramolecular C6 → C1 claisen condensation mediated by CHS, thus leading to resveratrol and naringenin, respectively.

Chalcone reductase (CHR) is an essential enzyme that participates in the metabolism of isoflavones in legumes [15]. It catalyzes the production of isoliquiritigenin (the key precursor of the 5-deoxyisoflavonoid), in conjunction with CHS. CHR generally belongs to the aldo-keto reductase 4 (AKR4) family, members of which harbor conserved catalytic tetrad consisting of Asp–Tyr–Lys–His [16]. Previously, it was generally accepted that CHR was exclusive to legumes. However, the discovery of DvAKR1, a member of the AKR13 subfamily identified from dahlias in the Asteraceae family, revealed significant divergence from legume CHRs [17]. This broke with conventional perception and suggested that plant CHR may have evolved independently of leguminous species.

F3H and FLS are the principal 2-oxoglutarate-dependent dioxygenase (2-ODD) enzymes involved in flavonol biosynthesis; the former converts flavanones to dihydroflavonols and the latter converts dihydroflavonols to flavonols. Their participation in the reaction typically necessitates 2-oxoglutarate and activated molecular oxygen as co-substrates, and Fe^2+^ and ascorbic acid as cofactors [18]. Phylogenetic analysis reveals that FLSs and F3Hs are categorized into two distinct clades and FLSs share a low level of sequence similarity with F3Hs (<35%) [19]. Nonetheless, both of them contain 2-oxoglutarate binding (RxS) and the ferrous ion binding sites (HxDxnH). Previous research indicated that certain FLSs are functionally promiscuous and exhibit additional F3H activity, such as OsFLS [20], CsFLS2 [21], and BnaFLS1 [22]. Due to the substrate competition between FLS and DFR (dihydroflavonol 4-reductase) [23], FLS is the key enzyme controlling flavonol metabolic flux. The overexpression of DoFLS1 in *Arabidopsis thaliana* could enhance flavonol accumulation while reducing anthocyanin levels and improving responses to abiotic stress [24].

There is a paucity of literature regarding the biosynthesis of stilbenoids and specific flavonoids in mulberry [25, 26]. Given that mulberry is abundant in distinctive 4′,6′-dihydroxylated flavonoids, we hypothesize the existence of an alternative parallel pathway commencing with 2,4-dihydroxycinnamic acid. 4-Coumarate: CoA ligase (4CL) could convert 2,4-dihydroxycinnamic acid into 2,4-dihydroxycinnamoyl-CoA, which can then undergo condensation with malonyl-CoA by CHS, followed by isomerization by CHI or spontaneous cyclization to form steppogenin, a 4′,6′-dihydroxylated flavanone. Steppogenin can undergo further modifications by prenyltransferase or cytochrome P450 to produce complex derivatives. Similarly, the biosynthesis of oxyresveratrol may necessitate catalysis by STS. Whether such functional CHS and STS enzymes are present in mulberry warrants further investigation. Therefore, we aimed to elucidate the biosynthetic pathways of flavonoids and stilbenoids in *M. notabilis* for facilitating the production and application of these bioactive components. In this study, two *Mn4CLs* (*Mn4CL1* and *Mn4CL2*), two PKSs (*MnCHS* and *MnSTS*), three *MnCHRs* (*MnCHR1–3*), and two 2-ODDs (*MnF3H* and *MnFLS*) genes were identified and functionally validated from *M. notabilis*. We speculate that steppogenin and oxyresveratrol were probably produced by the promiscuous CHS and STS from 2,4-dihydroxycinnamoyl-CoA, respectively. We have outlined a comprehensive biosynthetic pathway for flavonoids and stilbenoids in mulberry and provided new insights into the accumulation of bioactive components.

## Results

### Accumulation of flavonoids and stilbenoids in roots, stems, and leaves of *M. notabilis*

**Figure 2:**
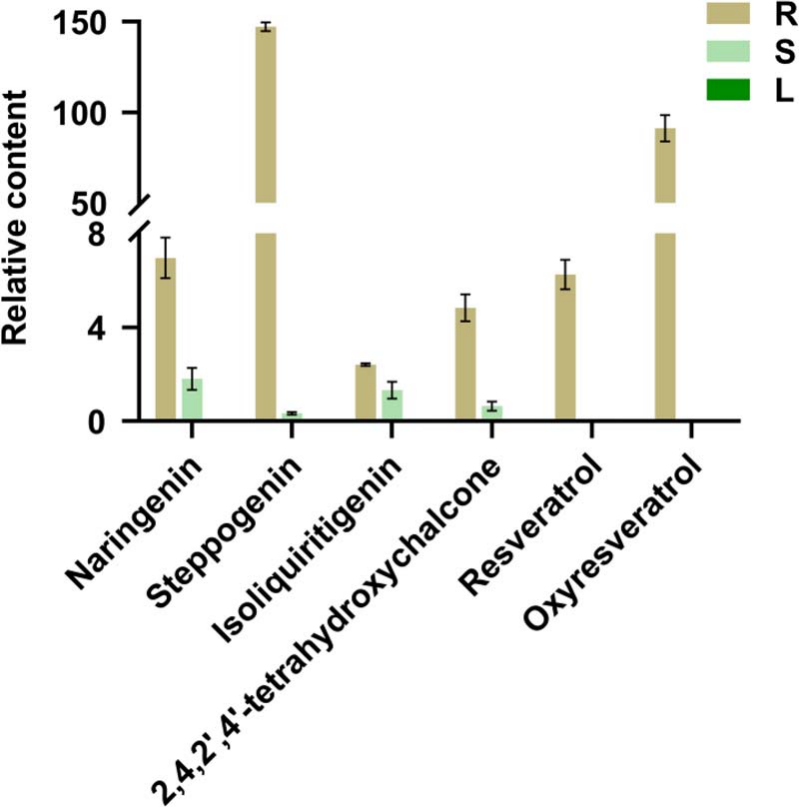
Accumulation of representative compounds in different tissues. The distribution of representative compounds in different tissues of *M. notabilis*, determined by LC–MS. R, root; S, stem; L, leaf. Three replications were performed for each analysis; error bars indicate the SD.

The distributions of representative flavonoid and stilbenoid metabolites in different plant tissues were analyzed through liquid chromatography–mass spectrometry (LC–MS), as illustrated in Fig. 2. Typical flavonoids naringenin, steppogenin, isoliquiritigenin, and 2,4,2′,4′-tetrahydroxychalcone were predominantly distributed in the roots, with lower contents present in the stems, while they were undetectable in the leaves. For the stilbenoids, resveratrol and oxyresveratrol were exclusively concentrated in the roots, with no accumulation in the stems or leaves. Among these compounds, steppogenin and oxyresveratrol were the most abundant, exceeding the levels of other metabolites by more than 100 times. These findings indicate a significant organizational differentiation in the distribution of metabolites within the plant.

### Functional characterization of the Mn4CLs from *M. notabilis*

**Figure 3:**
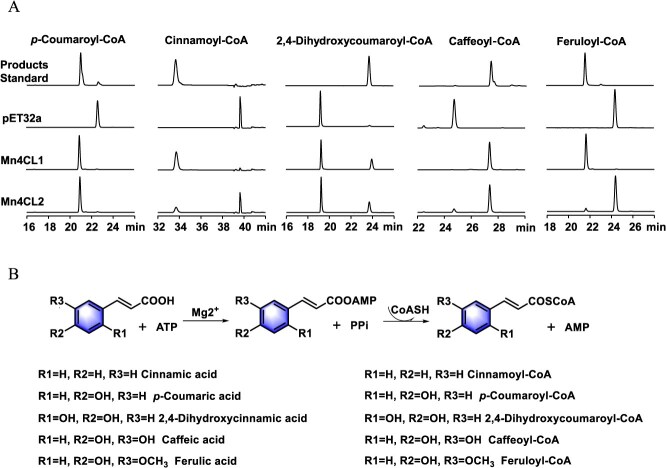
Enzymatic characterization of recombinant Mn4CL proteins. (A) The HPLC chromatograms of Mn4CLs’ *in vitro* enzyme activity with cinnamic acid, *p*-coumaric acid, 2,4-dihydroxycinnamic acid, caffeic acid, and ferulic acid as substrates. The protein expressed from the empty pET32a vector was used as the negative control and showed no detectable activity under identical conditions. (B) The different substrates and corresponding products catalyzed by Mn4CLs.

We searched the transcriptome database of *M. notabilis* using the keyword ‘4-coumarate: CoA ligase’; two possible 4CL genes were identified and named *Mn4CL1* and *Mn4CL2*, encoding 546 and 592 amino acids, respectively. A phylogenetic analysis based on Mn4CLs together with the previously identified 4CLs from other plants (Fig. S1) revealed that they were located in different branches of the evolutionary tree. Mn4CL1 was placed among the class I 4CLs, which were associated with lignin biosynthesis, whereas Mn4CL2 clustered with the class II 4CLs associated with the flavonoid biosynthesis. The sequence alignment demonstrated that both Mn4CL1 and Mn4CL2 contained conserved Box I (SSGTTGLPKGV) and Box II (GEICIRG) structural domains (Fig. S2). SDS-PAGE analysis confirmed the heterologous expression of the recombinant proteins (Fig. S3). The catalytic activities of recombinant Mn4CL proteins were assayed with a panel of common substrates (*p*-coumaric acid, cinnamic acid, caffeic acid, ferulic acid) and an additional untested substrate 2,4-dihydroxycinnamic acid. The high-performance liquid chromatography (HPLC) results indicated that Mn4CL1 was able to efficiently convert *p*-coumaric acid, 2,4-dihydroxycinnamic acid, cinnamic acid, caffeic acid, and ferulic acid into their corresponding CoA esters. Mn4CL2 could catalyze *p*-coumaric acid, 2,4-dihydroxycinnamic acid, and caffeic acid (Fig. 3A). Given that Mn4CL1 exhibited superior enzymatic activity, we selected Mn4CL1 as the candidate gene for subsequent pathway reconstruction in *Escherichia coli* BL21.

### Identification and functional validation of the MnCHS and MnSTS from *M. notabilis*

**Figure 4:**
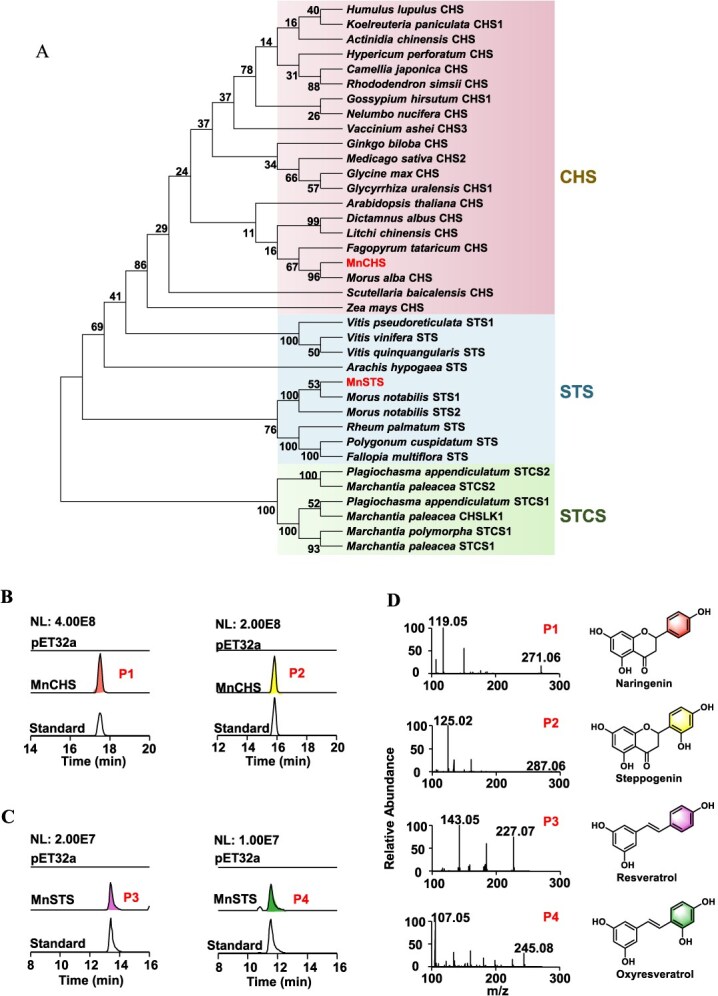
Phylogenetic tree and functional characterization of PKS. (A) Phylogenetic analysis of MnCHS, MnSTS, and other PKS proteins. The evolutionary tree is divided into three clusters (CHS, STS, and STCS), and candidate genes are MnCHS and MnSTS. (B) *In vitro* enzyme assays of MnCHS determined by LC–MS with *p*-coumaroyl-CoA and 2,4-dihydroxycinnamoyl-CoA as the substrates. (C) LC–MS results of MnSTS using *p*-coumaroyl-CoA and 2,4-dihydroxycinnamoyl-CoA as the substrates. (D) MS/MS fragmentation profile of the products P1, P2, P3, and P4.

Using ‘chalcone synthase’ and ‘stilbene synthase’ as the keywords, one putative CHS and one STS were selected and designated as MnCHS and MnSTS. The isolated MnCHS and MnSTS were identified with a full length of 1176 and 1200 bp, encoding 391 and 399 amino acid residues, respectively. Phylogenetic analysis showed that MnCHS and MnSTS were clustered within the chalcone synthase and stilbene synthase clades, respectively (Fig. 4A). Sequence alignments (Fig. S4) showed that MnCHS and MnSTS contained four conserved active site residues: Cys164, Phe215, His303, and Asn336 (with residue numbering corresponding to MsCHS2), especially the Cys–His–Asn triad, which is critical for substrate binding in type III PKS. Enzymatic assays were carried out using *p*-coumaroyl-CoA and 2,4-dihydroxycinnamoyl-CoA as the substrates. When *p*-coumaroyl-CoA was used as substrate, one product peak (P1) was observed in the LC–MS profile (Fig. 4B). By comparing with the standard and analyzing the MS spectrum data, the product generated by MnCHS was identified as naringenin. The initial product catalyzed by CHS is naringenin chalcone, which can spontaneously cyclize to form naringenin in the reaction solution [27]. When incubated with 2,4-dihydroxycinnamoyl-CoA, MnCHS led to the generation of another flavanone product. LC–MS analysis indicated this product (P2) exhibited an m/z value of 287.06 [M-H]^−^, with a retention time and fragmentation pattern identical to that of the standard steppogenin. To determine the enzymatic activity of MnSTS, an *in vitro* enzyme assay was conducted utilizing the purified recombinant protein. In the reaction with *p*-coumaroyl-CoA as the substrate, LC–MS chromatograms showed that MnSTS can catalyze the generation of resveratrol (Fig. 4C). Similarly, when MnSTS was incubated with 2,4-dihydroxycinnamoyl-CoA, one enzymatic peak was observed. The product peak was identified as oxyresveratrol, as it exhibited the same retention time and fragmentation ions as the standard (Fig. 4C). Previous investigations suggested that oxyresveratrol is probably derived from resveratrol through an oxidation process [28]. Here we successfully synthesized oxyresveratrol via direct catalysis using coenzyme A as the substrate, thus circumventing the involvement of CYP450 hydroxylase enzyme. Our findings present a novel approach for the synthesis of oxyresveratrol. Kinetic analysis revealed that MnCHS exhibited higher affinity and enhanced catalytic efficiency for *p*-coumaroyl-CoA compared to 2,4-dihydroxycinnamoyl-CoA (Table 1). In contrast, MnSTS preferred 2,4-dihydroxycinnamoyl-CoA as the substrate, demonstrating higher catalytic efficiency toward it than toward *p*-coumaroyl-CoA.

**Table 1:** The kinetic parameters of recombinant MnCHS and MnSTS

Enzyme	Substrate	*K*_m_ (μM)	*V*_max_ (nmol mg^−1^ min^−1^)	*K*_cat_ (min^−1^)	*K*_cat_/*K*_m_ (M^−1^ min^−1^)
MnCHS	*p*-Coumaroyl-CoA	6.44 ± 0.94	8.10 ± 0.33	0.49 ± 0.02	7.61 × 10^4^
	2,4-Dihydroxycinnamoyl-CoA	26.03 ± 3.78	10.03 ± 0.65	0.61 ± 0.04	2.33 × 10^4^
MnSTS	*p*-Coumaroyl-CoA	52.25 ± 7.94	13.41 ± 1.10	0.82 ± 0.07	1.57 × 10^4^
	2,4-Dihydroxycinnamoyl-CoA	26.34 ± 4.11	18.60 ± 1.23	1.13 ± 0.08	4.31 × 10^4^

### Functional characterization of the MnCHRs from *M. notabilis*

**Figure 5:**
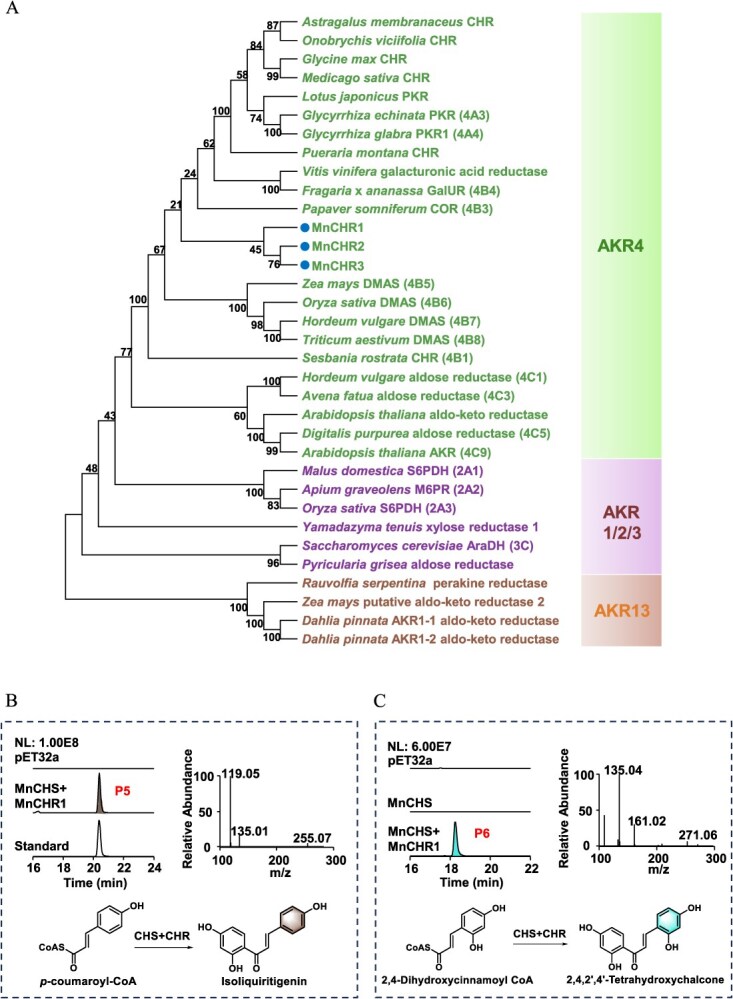
Phylogenetic tree and functional characterization of MnCHRs. (A) Phylogenetic analysis of MnCHRs and other AKRs superfamily proteins. Three candidate genes are marked with dots. LC–MS profiles of enzymatic reaction products generated from the co-incubation of MnCHS and MnCHR1 with *p*-coumaroyl-CoA (B) and 2,4-dihydroxycinnamoyl-CoA (C) as the substrates.

Phytochemical investigations have revealed that mulberry is capable of accumulating 6′-deoxychalcone compounds, the synthesis of which generally necessitates the participation of CHR. Consequently, we posited the existence of potential CHR genes related to 6′-deoxychalcone biosynthesis in mulberry. A thorough screening was conducted utilizing the AKR domain PF00248 as a query within the transcriptome database, resulting in the identification of three putative CHR genes: *MnCHR1*, *MnCHR2*, and *MnCHR3*. Phylogenetic analysis was then performed using these putative CHRs and individual representatives from other AKR families. The three MnCHRs form a separate clade within the AKR4B subfamily, while being phylogenetically proximate to the AKR4A subfamily that encompasses legume CHRs (Fig. 5A). The pairwise sequence similarity among three MnCHR genes is approximately 60%; they are paralogs. Sequence comparison revealed that MnCHR1, MnCHR2, and MnCHR3 contained a conserved catalytic tetrad comprising Asp53, Tyr58, Lys87, and His120 (with residue numbering corresponding to MsCHR), except for MnCHR2 lacking K87 (Fig. S5). The enzymatic activity of CHRs was assessed through co-incubation with MnCHS *in vitro*. LC–MS profiles (Fig. 5B) displayed that MnCHS and MnCHRs could catalyze *p*-coumaroyl-CoA to produce isoliquiritigenin, with MnCHR1 exhibiting the highest activity (Fig. S6). Therefore, MnCHR1 was chosen as the candidate gene for further experiments. Furthermore, when 2,4-dihydroxycinnamoyl-CoA was used as the substrate, only MnCHR1 catalyzed the formation of 2,4,2′,4′-tetrahydroxychalcone (Fig. 5C). LC–MS analysis revealed that P6 exhibited an m/z value of 271.06 [M-H]^−^ and a fragmentation pattern consistent with the reported literature [29].

**Figure 6:**
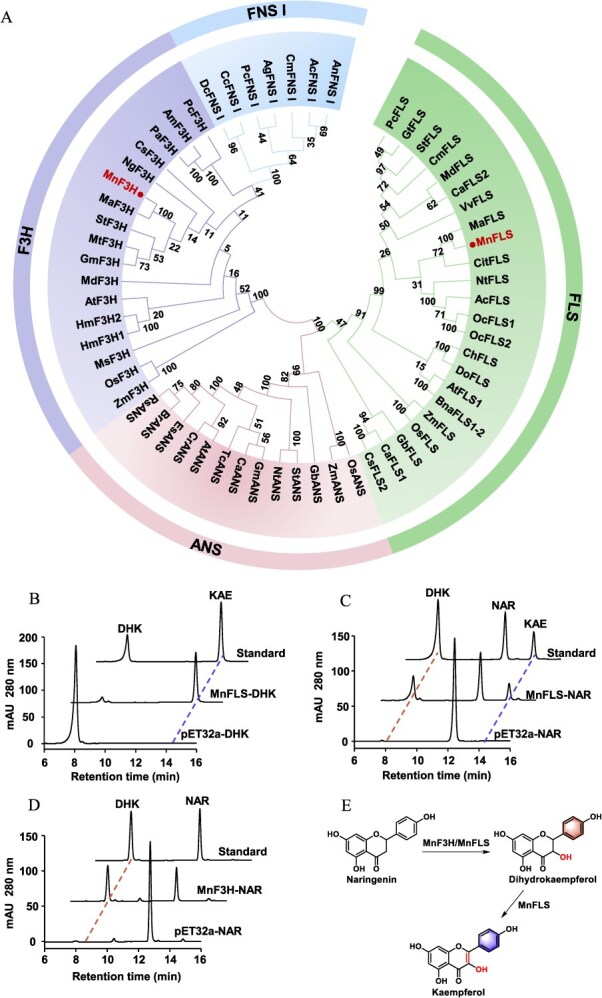
Identification and functional characterization of MnFLS and MnF3H. (A) Phylogenetic analysis of MnFLS and MnF3H with other plants 2-ODDs. FNS I, flavone synthase I; ANS, anthocyanidin synthase. MnFLS and MnF3H are marked with dots. Four clusters (FNS I, F3H, ANS, and FLS) are indicated in the evolutionary tree. HPLC chromatogram of DHK (B) and NAR (C) catalyzed by recombinant MnFLS, and NAR (D) catalyzed by MnF3H. (E) Reactions catalyzed by MnFLS and MnF3H. DHK, dihydrokaempferol; NAR, naringenin; KAE, kaempferol.

### Identification and functional characterization of the MnF3H and MnFLS

Two 2-ODD sequences MnF3H and MnFLS were obtained from *M. notabilis* transcriptome using the search terms ‘flavonol synthase/flavanone 3-hydroxylase’. Their complete sequences encompass open reading frames of 1098 and 1008 bp, encoding 365 and 335 amino acids, respectively. Sequence alignment showed that both sequences contain conserved motifs characteristic of 2-ODD enzymes: the ferrous ion binding (HxDxnH) and 2-OG binding (RxS) residues (Fig. S8). In addition, MnFLS harbored unique motifs specific to FLS, ‘PxxxIRxxxEQP’ and ‘SxxTxLVP’, distinguishing it from other 2-ODD proteins [30]. Phylogenetic tree was constructed to reveal the evolutionary relationships between MnFLS/MnF3H and other 2-ODD sequences from various plants. Based on the phylogenetic analysis, MnFLS and MnF3H were classified into the FLS and F3H gene families (Fig. 6A). To verify the activity of the recombinant MnFLS, dihydrokaempferol was utilized as a substrate. The results of the *in vitro* enzyme activity demonstrated that MnFLS effectively catalyzed the conversion of dihydrokaempferol to kaempferol, confirming its role as a typical flavonol synthase (Fig. 6B). Additionally, MnFLS could convert naringenin into dihydrokaempferol and subsequently dihydrokaempferol into kaempferol, indicating that MnFLS exhibits both F3H and FLS activities (Fig. 6C). Thus, MnFLS is a bifunctional 2-ODD enzyme, demonstrating dual catalytic capabilities for mediating two reactions. Kinetic experiments showed that MnFLS had stronger affinity for naringenin (*K*_m_ = 40.19 ± 4.92 μM) than for dihydrokaempferol (*K*_m_ = 62.27 ± 15.51 μM). The *K*_cat_/*K*_m_ values of MnFLS toward dihydrokaempferol and naringenin were 1.25 × 10^5^ and 4.69 × 10^4^ M^−1^ min^−1^, which indicated that MnFLS possessed higher catalytic efficiency for dihydrokaempferol (Table 2). Further investigations into the catalytic activity of MnFLS on five additional flavanones revealed that, except for eriodictyol, which yielded no detectable product, the remaining four substrates were successfully converted into their corresponding dihydroflavonols and subsequently into flavonols (Fig. S9). In parallel, the catalytic function of MnF3H was explored with six flavanone substrates (Fig. 6D and Fig. S10). MnF3H effectively catalyzed them to generate corresponding dihydroflavonols and was thereby validated as a bona fide F3H.

**Table 2:** The kinetic parameters of MnFLS for different substrates

Enzyme	Substrate	*K*_m_ (μM)	*V*_max_ (nmol mg^−1^ min^−1^)	*K*_cat_ (min^−1^)	*K*_cat_/*K*_m_ (M^−1^ min^−1^)
MnFLS	DHK	62.27 ± 15.51	138.81 ± 11.83	7.77 ± 0.66	1.25 × 10^5^
	NAR	40.19 ± 4.92	33.69 ± 1.61	1.89 ± 0.09	4.69 × 10^4^

**Figure 7:**
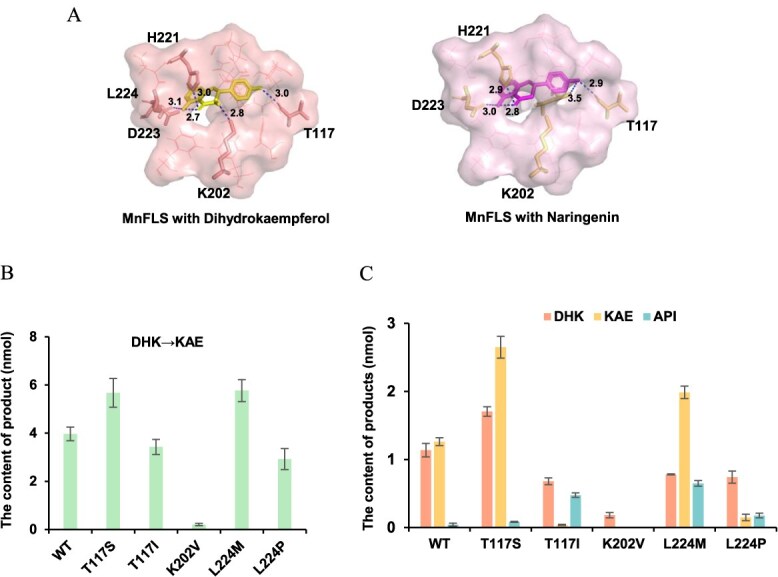
Characterization of key residues associated with the catalytic activity of MnFLS by molecular docking and site-directed mutagenesis. (A) Molecular docking results of MnFLS with dihydrokaempferol and naringenin. Dashed lines: H bond interactions. Enzymatic activity variations of site-directed MnFLS mutants when DHK (B) and NAR (C) were used as substrates, respectively. DHK, dihydrokaempferol; NAR, naringenin; KAE, kaempferol; API, apigenin. Results are means ± SD, *n* = 3.

**Figure 8:**
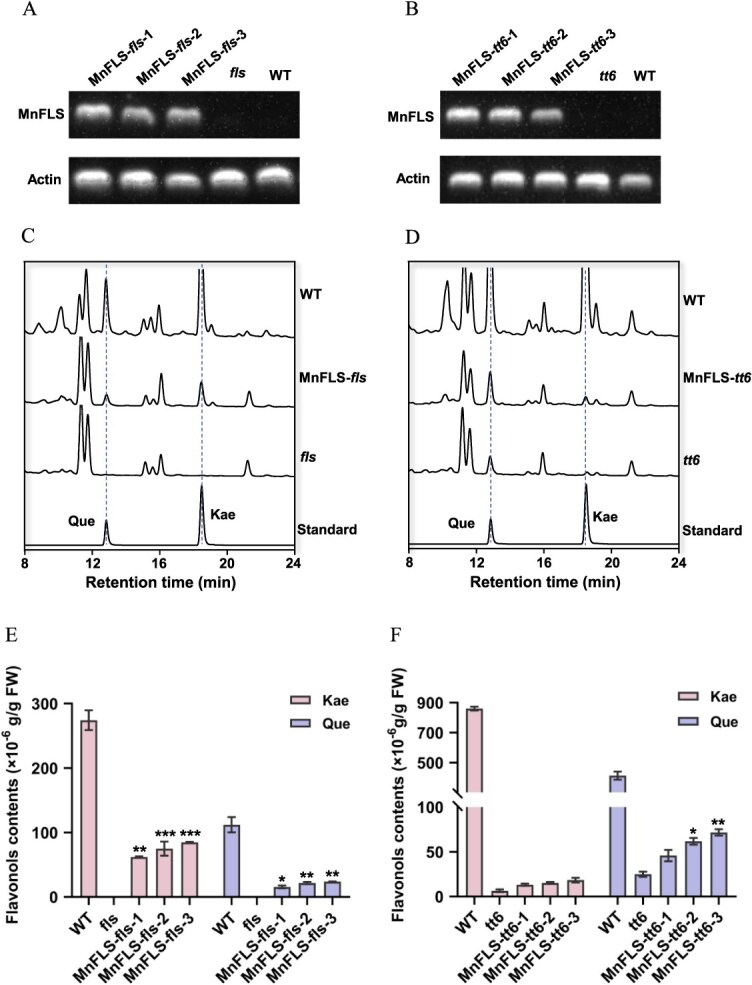
The heterologous expression of MnFLS in *fls* and *tt6* mutants and accumulation of flavonols in WT, mutant, and transgenic lines. (A and B) RT-PCR expression analysis of MnFLS in the transgenic lines, mutant, and WT. (C and D) Representative HPLC profiles of methanolic extracts of WT, *fls*/*tt6* mutant, and MnFLS-*fls*/*tt6* transgenic seedlings. (E and F) Comparison of the flavonol contents in WT, *fls*/*tt6* mutants, and transgenic *Arabidopsis* seedlings. FW, Fresh weight; Que, quercetin; Kae, kaempferol. Data are shown in the form of mean ± SD of three replicates. ^*^P < 0.05, ^**^P < 0.01, ^***^P < 0.001.

In order to investigate the critical residues that contribute to the function of MnFLS, we carried out molecular docking and site-directed mutagenesis with substrates naringenin and dihydrokaempferol. Through molecular docking, we targeted several key residues Thr117, Lys202, and Met224, which can form interactions with the substrates. Subsequently, we constructed mutants of these residues. The residues Thr117 can form hydrogen bonding interactions with substrates. Substituting Thr117 with Ser without a methyl group, the mutant T117S resulted in significant enhancement of activity for both naringenin and dihydrokaempferol as substrates (Fig. 7). The elimination of the methyl group extended the substrate binding pocket. Conversely, replacing the Thr117 with Ile led to reduced activity. The presence of methyl groups introduced steric hindrance effect, adversely affecting the binding of the protein to the substrate. Lys202 emerged as a pivotal residue for substrate binding; notably, the mutant K202V suffered an almost complete loss of enzymatic function. The variant L224M facilitated the conversion of dihydrokaempferol to kaempferol, promoted an increased synthesis of dihydrokaempferol and kaempferol from naringenin, and additionally produced minor quantities of apigenin. This suggests that the mutant appears to have higher FNS I activity in comparison to the wild-type enzyme.

**Figure 9:**
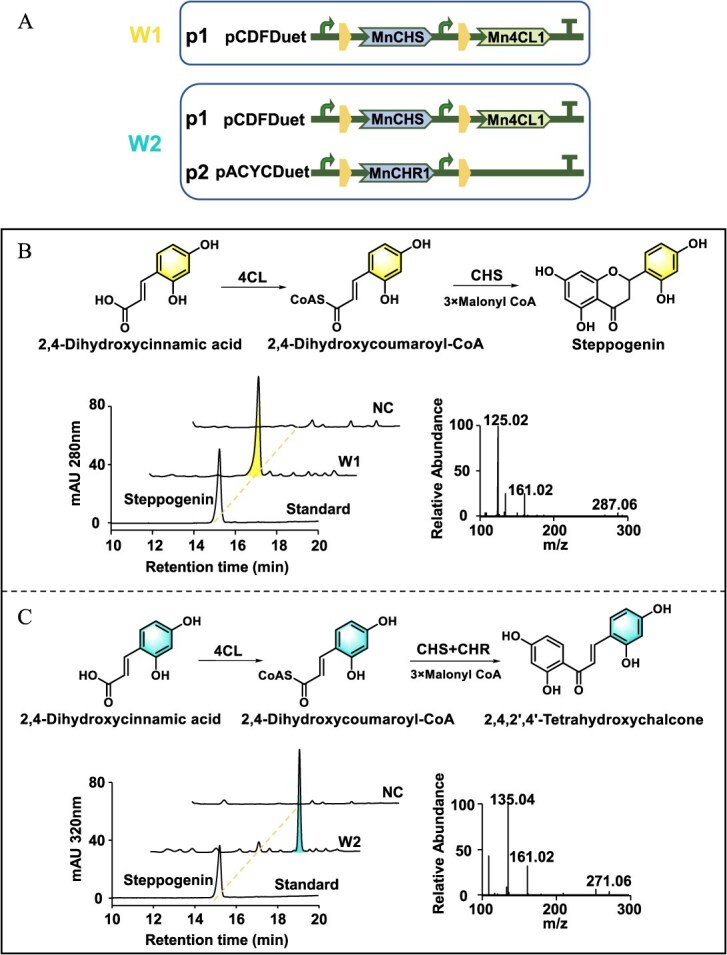
Production of flavonoids through bioconversion in *E. coli*. (A) Schematic diagrams of two recombinant *E. coli* strains W1 and W2, constructed with the genes Mn4CL1, MnCHS, and MnCHR1. Biosynthetic pathway for flavonoid production from 2,4-dihydroxycinnamic acid and identification of products by recombinant strains W1 (B) and W2 (C).

### Heterologous expression of MnFLS increased flavonol content in transgenic *A. thaliana*

To investigate the *in vivo* role of MnFLS in plants, the gene was overexpressed in the *Arabidopsis* mutants *fls* (without FLS activity) and *tt6* (without F3H activity). The T3 generation of positive transgenic strains was successfully obtained after screening for hygromycin resistance. The transcripts of the overexpressed MnFLS in both *fls* and *tt6* were detected in the resulting T3 generation of transgenic plants (Fig. 8A and B). The accumulation of flavonols was then analyzed by HPLC in the wild-type (WT), mutant, and transgenic *Arabidopsis* lines. Acid-hydrolyzed flavonoid extracts from MnFLS-*fls* transgenic seedlings obviously accumulated kaempferol and quercetin, which were absent in the *fls* mutant (Fig. 8C and E). This observation hinted that MnFLS can indeed exert FLS activity in plants, prompting the conversion of dihydroflavonols into flavonols. Thus, MnFLS could compensate for the absence of the FLS gene in *A. thaliana fls* mutant, diverting the metabolic flow from anthocyanins to flavonols. Compared with the *tt6* mutant, MnFLS-*tt6* transgenic seedlings demonstrated slight increase in the accumulation of kaempferol and quercetin (Fig. 8D and F). This may be due to the weak F3H activity exerted by MnFLS in the plant, which failed to efficiently convert the accumulated flavanones in *tt6* into dihydroflavonols and flavonols. Despite the fact that MnFLS exhibited bifunctionality of FLS and F3H *in vitro*, its predominant role in plants is that of FLS, with minor F3H activity.

### Gene expression analysis of *M. notabilis*

The expression of key genes involved in the biosynthesis of flavonoid and stilbenoid compounds was examined by qPCR experiments (Fig. S11A). Among these candidate genes, the highest transcript levels of *Mn4CL1*, *MnCHR2*, and *MnCHR3* were found in the leaves or stems. Conversely, *Mn4CL2*, *MnCHS*, *MnSTS*, and *MnCHR1* were expressed primarily in the roots, with comparatively lower expression in stems and leaves. The tissue expression patterns of *Mn4CL2*, *MnCHS*, *MnSTS*, and *MnCHR1* were consistent with the accumulation of aforementioned metabolites. Moreover, we performed correlation analysis to further support the above results (Fig. S11B). It indicated that the accumulation of six representative compounds was significantly correlated with the expressions of *Mn4CL2*, *MnCHS*, *MnSTS*, and *MnCHR1* genes. These results suggested that *Mn4CL2*, *MnCHS*, *MnSTS*, and *MnCHR1* might play a crucial role in the accumulation of flavonoids and stilbenoids.

### Construction of the heterologous flavonoid synthetic pathway in *E. coli*

With the aim of achieving heterologous biosynthesis of the characteristic compounds, we constructed engineered strains of *E. coli*, as illustrated in Fig. 9A. The strain W1 harboring the plasmid pCDFDuet-MnCHS-Mn4CL1 was supplemented with *p*-coumaric acid and 2,4-dihydroxycinnamic acid. As we expected, the addition of 2,4-dihydroxycinnamic acid to the mediums can produce significant quantities of steppogenin compared with the negative control (Fig. 9B). However, no naringenin was detected when *p*-coumaric acid was introduced into the fermentation broth. We speculated that the *E. coli* may have metabolized the substrate *p*-coumaric acid or that the resultant naringenin flowed into other metabolic pathways. We also incorporated pCDFDuet-MnCHS-Mn4CL1 and pACYCDuet-MnCHR1 into *E. coli* to obtain strain W2. The same situation was observed in feeding trials with strain W2. When utilizing *p*-coumaric acid as the precursor for the biotransformation, no expected products were generated. However, the precursor 2,4-dihydroxycinnamic acid can be biotransformed to a certain quantity of 2,4,2′,4′-tetrahydroxychalcone (Fig. 9C). It has been reported that intracellular concentration of malonyl-CoA is essential for the efficient production of malonyl-CoA derivatives. However, *E. coli* exhibits notably low level of cellular malonyl-CoA, which constrains the large-scale production of valuable malonyl-CoA derivatives. Therefore, employing yeast or genetically modified *E. coli* to enhance malonyl-CoA levels may represent promising strategies [31].

## Discussion

As a traditional plant with historical usage spanning thousands of years, the mulberry holds a vital position in various sectors, including the silk industry, food, medicine, and cosmetics. In particular, its medicinal value suggests promising development prospects. Phytochemical studies have revealed that mulberry contains distinctive 4′,6′-dihydroxylated flavonoids, as well as stilbenoids. As precursor substances, they can be subsequently converted into more complex derivatives by prenyltransferase and cytochrome P450. For instance, prenylated flavone morusin has been appreciated for versatile salutary effects, including antioxidant, antitumor, anti-inflammatory, and anti-hyperglycemic activities [32]. Considering that versatile medicinal activities are intimately linked to unusual constituents, it is important to determine whether a parallel biosynthetic pathway exists in *M. notabilis* that utilizes 2,4-dihydroxycinnamic acid as a substrate to produce these flavonoids. Here, we cloned two Mn4CLs, two PKSs, three MnCHRs, and two 2-ODDs from mulberry, and verified their catalytic activity *in vitro* and *in vivo*. Eventually, we proposed a possible new biosynthetic pathway for flavonoids and stilbenoids in mulberry (Fig. S12).

### MnCHS and MnSTS may participate in two parallel biosynthetic pathways in *M. notabilis*

The biosynthesis of steppogenin is likely dependent on 2,4-dihydroxycinnamic acid as a substrate and involves sequential catalysis of two key enzymes 4CL and CHS. In the present investigation, Mn4CL1 and Mn4CL2 can catalyze not only common *p*-coumaric acid but also 2,4-dihydroxycinnamic acid. MnCHS catalyzed the conversion of 2,4-dihydroxycinnamoyl-CoA into the chalcone backbone, which underwent auto-cyclization to yield steppogenin. This process closely resembles the synthesis of naringenin. MnCHS played an important role in the biosynthesis of flavanone naringenin and steppogenin in mulberry. This indicated the existence of an additional metabolic pathway associated with 2,4-dihydroxycinnamic acid. Moreover, this pathway promotes the accumulation of 4′,6′-dihydroxylated flavonoids specific to mulberry. Similarly, MnSTS catalyzed the conversion of *p*-coumaroyl-CoA and 2,4-dihydroxycinnamoyl-CoA into resveratrol and oxyresveratrol, respectively. 2,4-Dihydroxycinnamoyl-CoA was employed to facilitate the direct one-step synthesis of oxyresveratrol, effectively bypassing the involvement of CYP450. This research lays the groundwork for the industrial biosynthesis of oxyresveratrol in the future. However, whether CYP450 enzymes participate in oxyresveratrol biosynthesis remains to be investigated.

### *MnCHR1* is the key gene responsible for 6′-deoxychalcone biosynthesis in mulberry

The chalcones and their derivatives hold significant relevance due to their extensive therapeutic potential [33]. Mulberry is rich in chalcone compounds, including 6′-deoxychalcone, which can exist in free form as well as combine with other compounds to form Diels-Alder adducts. The synthesis of 6′-deoxychalcone requires the participation of CHR. Our study discovered three CHR genes *MnCHR1*–*3* and examined their influence on the 6′-deoxychalcone accumulation. MnCHR1 showed the best activity to yield isoliquiritigenin or 2,4,2′,4′-tetrahydroxychalcone by collaborating with MnCHS. Moreover, its expression was correlated with 6′-deoxychalcone accumulation in mulberry. This marks the first identification of CHR genes from the Moraceae family, accompanied by functional characterization. Moreover, we designed an artificial biosynthetic pathway starting from 2,4-dihydroxycinnamic acid, which enabled efficient production of 2,4,2′,4′-tetrahydroxychalcone in *E. coli* strain W2 (Fig. 9). This developed biosynthetic pathway holds promise for application in large-scale fermentation production of 6′-deoxychalcones.

NADPH-dependent CHR activity was initially reported in soybean cell suspension cultures [34]. Subsequently, more CHR genes were identified in other legumes such as *Sesbania rostrata* [35], *M. sativa* [36], and *Astragalus membranaceus* [37], and they existed as a multigene family. The legume CHRs generally belong to the AKR4A subfamily, except for *S. rostrata* CHR, which is a member of the AKR4B subfamily. Generally speaking, nonleguminous CHR is relatively scarce in the plant kingdom and has been reported only in dahlias from Asteraceae family [17]. The MnCHRs identified in this study belong to AKR4B subfamily, and they were phylogenetically close to AKR4A CHRs from legumes (Fig. 5A). MnCHR proteins shared 45%–50% amino acid sequence identity with *Glycine max* CHR protein (AHG25321.1). It was indicated that the CHRs from Moraceae and legume family probably have a shared evolutionary origin.

### Consistency between gene expression patterns and metabolite accumulation

Figuring out the spatial distribution of flavonoid and stilbenoid components can guide the rational utilization of different tissues for diverse medicinal applications. The content of flavonoids and stilbenoids showed organ-specificity, and these compounds were prominently enriched in the roots (Fig. 2). Organization expression of the genes revealed that most of related genes were expressed preferentially in the roots (Fig. S11A). Among the CHR homologs, *MnCHR1* exhibited the strongest spatial correlation with 6′-deoxychalcones accumulation and displayed the best catalytic activity. It can be concluded that *Mn4CL2*, *MnCHS*, *MnSTS*, and *MnCHR1* were expressed in a pattern coincident with the accumulation of flavonoids and stilbenoids. These findings explained the high accumulation of metabolites in roots. Combined with the enzymatic validation results, there may exist a metabolic pathway related to 2,4-dihydroxycinnamoyl-CoA in mulberry, contributing to the accumulation of unique 4′,6′-dihydroxylated flavonoids.

### Functional exploration of the MnFLS

In addition to the presence of the characteristic flavonoids mentioned above, flavonols are also present in mulberry. Here, we demonstrated two key 2-ODD genes *MnFLS* and *MnF3H* that are essential for flavonol synthesis. Although MnFLS is classified within the FLS branch in the phylogenetic tree (Fig. 6A), it performed both FLS and F3H catalytic activities *in vitro*. To validate the function of MnFLS in plants, ectopic expression of MnFLS was conducted in *Arabidopsis fls* and *tt6* mutants. The levels of kaempferol and quercetin were noticeably increased in the MnFLS-*fls* transgenic lines, although they did not reach the levels observed in wild-type plants. The incomplete restoration of flavonol levels in MnFLS-*fls* lines could be attributed to suboptimal expression driven by the heterologous 35S promoter, and it is a normal phenomenon. Although CaMV 35S is an efficient promoter widely used in dicots, its efficiency may be lower than that of *Arabidopsis* endogenous promoter in some cases. In the case of the MnFLS-*tt6* transgenic lines, there was only a marginal increase in flavonol contents compared to the *tt6* line. In summary, *in planta* studies via mutant *Arabidopsis* successfully confirmed that bifunctional MnFLS predominantly performs FLS function in plants, with weak F3H activity. Prior studies found that several FLS enzymes OsFLS [20], CaFLS2 [38], and BnaFLS1 [22] could catalyze naringenin into dihydrokaempferol and dihydrokaempferol into kaempferol, indicating that these enzymes showed both FLS and F3H activities *in vitro*. Overexpressing BnaFLS1 in *A. thaliana f3h* and *ans/fls1* mutants can facilitate the accumulation of flavonols in mutants and restore their phenotype to wild type through flavonol staining of young seedlings and HPTLC analysis. However, there was no quantitative analysis of flavonol contents. F3H, FLS, FNS I, and ANS are the four principal 2-ODD enzymes in the flavonoid pathway. Evolutionary phylogenetic analysis showed that FNS I and F3H belong to the same clade, whereas FLS and ANS are situated in another clade (Fig. 6A). MnFLS and MnF3H exhibit extremely low amino acid sequence similarity (26%), and they are phylogenetically distant from each other. Nevertheless, MnFLS retains partial F3H activity. It has been proposed that FLS evolved via divergence from F3H [39]. Therefore, it is probable that FLS may have retained certain specific active sites of F3H during the course of evolution, hence displaying partial F3H activity. F3H harbors conserved residues critical for 2-ODD enzymes, but F3H-specific residues or motifs remain unclear. Thus, further investigations are warranted to delineate the molecular basis underlying the functional divergence of these enzymes in the future.

## Conclusion

In conclusion, our study delineated the biosynthetic pathway of distinctive flavonoids and stilbenoids in *M. notabilis*. Based on the results of enzymatic assays and the correlation patterns between flavonoid and stilbenoid concentrations and gene expression across various tissues, we hypothesize that Mn4CL2 catalyzes the conversion of 2,4-dihydroxycinnamic acid to 2,4-dihydroxycinnamoyl-CoA. This compound may subsequently condense with malonyl-CoA through the action of either MnCHS or MnSTS, leading to the formation of steppogenin or oxyresveratrol. Notably, we also identified CHRs from the Moraceae family for the first time, thereby broadening the known sources of CHRs beyond the legume family. Furthermore, we demonstrated that MnFLS predominantly exhibited FLS activity *in planta* through ectopic expression in the mutant *Arabidopsis*, although it also displayed F3H activity in the *in vitro* enzyme assay. A comprehensive analysis of the metabolic pathways for flavonoids and stilbenoids in mulberry offers significant insights for the large-scale biosynthesis of these unique compounds in microbial cell factories by constructing engineered *E. coli* or yeast.

## Materials and methods

### Plant materials and reagents

*Morus notabilis* used in this study was obtained from the Shandong University, Jinan, China. Upon collection, the samples were immediately frozen in liquid nitrogen and stored in an ultra-low temperature refrigerator at −80°C for subsequent experiments. *A. thaliana* was grown at 22°C under a 16-h light/8-h dark photoperiod. All commercially available compounds used in this study were purchased from Chengdu Must Bio-technology (Chengdu, China). The solvents used for HPLC and LC–MS were all analytical grade. *P*-Coumaroyl-CoA and 2,4-dihydroxycinnamoyl-CoA were synthesized referring to a previously reported method [40].

### Transcriptome sequencing, *de novo* assembly, and annotation

The mRNA library preparation was performed using Optimal Dual-mode mRNA Library Prep Kit (BGI-Shenzhen, China). The libraries were sequenced (PE150 bases reads) on the DNBSEQ-T7 platform (BGI-Shenzhen). The raw data were filtered with SOAPnuke (v1.4.0); afterward, clean reads were obtained and stored in FASTQ format. The clean reads were *de novo* assembled using Trinity (v2.0.6), and the assembly quality was assessed by BUSCO (v5.4.3). Annotating assembled Unigene with seven major functional databases (KEGG, GO, NR, NT, SwissProt, Pfam, and KOG).

### Compositional profiling of different tissues in *M. notabilis*

Different tissue samples were lyophilized and ground into powder. Fifty milligrams of powder was extracted with 600 μL of 80% methanol by sonication for 1 h in an ice water bath. Then, the samples were centrifuged at 12 000*g* for 20 min and supernatants were collected. An equal volume of 2 N HCl was added and the sample was heated in a water bath at 70°C for 40 min. Upon completion of the acid hydrolysis, an equal volume of ethyl acetate was added to extract the products twice, and the organic phase volatilized completely. The residues were dissolved with 80% methanol and filtered through a 0.22-μm microporous filter in preparation for LC–MS analysis (analytical method was the same as mentioned below).

### Identification, sequence alignment, and phylogenetic analysis of candidate genes

Related genes were searched in transcriptome data of *M. notabilis* (PRJNA1215339) and analyzed through NCBI. Using ‘4-coumarate: CoA ligase’ as a search term, two possible genes were identified from the transcriptomic database of *M. notabilis* and designated as *Mn4CL1* and *Mn4CL2*. Moreover, one putative CHS and one STS were screened using the keywords ‘chalcone synthase’ and ‘stilbene synthase’, and they were named as *MnCHS* and *MnSTS*. A comprehensive screening was conducted utilizing the AKR domain ‘PF00248’ as a query to identify potential CHR genes. As a result, three CHR genes were screened, namely *MnCHR1*, *MnCHR2*, and *MnCHR3*. In addition, two candidate genes, *MnFLS* and *MnF3H*, were identified based on the keywords ‘flavonol synthase/flavanone 3-hydroxylase’. Phylogenetic trees were constructed with the deduced amino acid sequences using a neighbor-joining method of MEGA v5.0.1 software (https://www.megasoftware.net) based on bootstrap analysis with 1000 replicates. All the accession numbers of the amino acid sequences used to construct the phylogenetic trees in this article were provided in Tables S1–S4. The sequence alignments of putative genes were performed using MEGA v5.0.1 software, and the alignment results were presented using the online tool Espript 3.0 (https://espript.ibcp.fr/ESPript/ESPript/index.php) [41].

### Gene cloning, heterologous expression, and protein purification

The total RNA was extracted from the mulberry leaves using the cetyltrimethylammonium bromide method. The isolated RNA was converted to cDNA using a PrimerScriptRT Master Mix kit (including gDNA eraser) (Takara, Kyoto, Japan) following the manufacturer’s instructions. The obtained cDNA served as a template to amplify the open reading frames (ORFs) of the candidate genes (*Mn4CL1* and *Mn4CL2*, *MnCHS* and *MnSTS*, *MnCHR1–3*, *MnFLS* and *MnF3H*). The amplified fragments were digested with corresponding restriction endonucleases and ligated into the pET32a vector. The primers used for gene cloning are detailed in Table S5. Recombinant plasmids harboring the candidate genes and empty pET32a plasmid were transformed into *E. coli* BL21 (DE3) competent cells (Novagen) for the prokaryotic expression. The recombinant proteins were expressed and purified according to reported protocols [42]. The purified proteins were analyzed using SDS-PAGE (Fig. S3).

### Enzymatic assays of Mn4CLs

The *in vitro* enzymatic activity of Mn4CLs was assayed by reaction at 30°C for 1 h in 200 μl. The reaction system comprised 200 mM Tris–HCl buffer (pH 7.5), 5 mM MgCl_2_, 5 mM ATP, 300 μM CoA-Li, and 200 μM substrate (*p*-coumaric acid, cinnamic acid, caffeic acid, ferulic acid, and 2,4-dihydroxycinnamic acid), along with 20 μg of purified recombinant protein. The reaction mixture was then boiled at 100°C for 5 min and centrifuged at 13 000 rpm for 20 min. The supernatant was analyzed by HPLC using a reverse-phase C18 column (XD-C18, 5 μm, 4.6 × 250 mm). The procedure of HPLC adheres to methodologies established in prior research [43].

### *In vitro* functional validation of MnCHS, MnSTS, and MnCHRs

Enzymatic reactions of MnCHS and MnSTS contained 100 mM potassium phosphate buffer (pH 7.5), 50 μM starter-CoA (*p*-coumaroyl-CoA and 2,4-dihydroxycinnamoyl-CoA), 100 μM malonyl-CoA, and 15 μg of purified recombinant protein. The protein expressed by the empty pET32a served as a negative control. Two hundred microliters of the above mixture was incubated at 35°C for 1 h, followed by extracting twice with an equal volume of ethyl acetate. The ethyl acetate phase was evaporated completely, and 100 μL of 80% methanol was added to dissolve the sample. The kinetic experiments were carried out according to the methods described in the reference [12]. The kinetic parameters (*K*_m_ and *V*_max_) were determined by nonlinear regression analysis of the Michaelis–Menten equation using GraphPad Prism 9.5.0. To verify the catalytic activity of MnCHRs, they were co-incubated with MnCHS at 35°C for 2 h. Based on the reaction system of MnCHS, additional 1 mM NADPH and 40-μg purified recombinant MnCHR protein were added. The reaction procedure was consistent with that of MnCHS. The processed samples were centrifuged and the supernatants were analyzed using LC–MS instrument (Thermo Fisher, USA) equipped with a C18 Hypersil Gold column (3 μm, 2.1 × 100 mm). The mobile phase comprised methanol (B) and 0.1% (v/v) formic acid water (D). The gradient conditions were as follows: 10% B/90% D at 0.0 min, 65% B/35% D at 25.0 min, 95% B/5% D at 26.0 min, and 10% B/90% D at 29.0 min, with a flow rate maintained at 0.3 ml min^−1^. Regarding the identification of the products, the theoretical m/z value of the target product is retrieved, and the retention time and fragmentation pattern are compared with those of the standard compound.

### Catalytic activity of Mn2-ODDs

Mn2-ODDs activity was evaluated by incubating at 30°C for 1 h in a 100-μl reaction system containing 15-μg recombinant protein, 50 mM Tris–HCl (pH 7.0), 0.64 mM 2-oxoglutaric acid, 1 mM ascorbate, 0.1 mM FeSO_4_, and 100 μM substrate (dihydrokaempferol, naringenin, pinocembrin, liquiritigenin, eriodictyol, hesperetin, and homoeriodictyol). The protein expressed from the empty pET32a plasmid was used as the negative control. An equal volume of ethyl acetate was added to extract the reaction products twice and centrifuged at 13 000 rpm for 5 min. The organic phase was evaporated and the residues were dissolved in 100-μl methanol for HPLC analysis using an Agilent Zorbax SB-C18 column (150 mm × 4.6 mm, 5-μm pore size) at a flow rate of 0.8 ml min^−1^. The mobile phase consisted of methanol (A) and 0.1% (v/v) formic acid water (B). The gradient conditions were as follows: 0–20 min, 30%–80% A; 20.1–25 min, 100% A; 25.1–30 min, 30% A. Kinetic studies of the recombinant MnFLS were conducted according to the previous report [44]. The kinetic parameters (*K*_m_ and *V*_max_) were determined by nonlinear regression analysis of the Michaelis–Menten equation using GraphPad Prism 9.5.0.

### De novo modeling and site-directed mutagenesis of MnFLS

The protein structure of MnFLS was predicted by AlphaFold Server (https://alphafoldserver.com/) [45]. The molecular interaction docking analysis of MnFLS with naringenin and dihydrokaempferol was performed separately using Schrodinger Suites software (https://www.schrodinger.com/). The active pockets were constructed and the key amino acid sites were identified using visual PyMOL v2.5.2 software (https://pymol.org). The pET32a (+)-MnFLS was used as a template for targeted mutagenesis using mutation primers. Mutation primers were designed by Primer X Online website (https://www.bioinformatics.org/primerx/index.htm), as shown in Table S8. Mutations were performed using Overlap Extension PCR, with the detailed procedure referenced from a prior report [46]. The expression, purification, and enzyme assays of the mutants were the same as wild-type Mn2-ODDs.

### Heterologous expression of MnFLS in *A. thaliana*

MnFLS ORF was PCR-amplified using the attB-MnFLS-F/R primer pairs listed in Supplemental Table S7. The MnFLS-pMpGWB111 vector was constructed following previously established protocols, after which it was introduced into the *Agrobacterium tumefaciens* strain GV3101 [47]. Subsequently, the resulting bacteria were transformed into *A. thaliana* mutants *tt6* and *fls* via the floral dip method [48]. The positive transgenic seedlings were selected by sowing *Arabidopsis* seeds on 1/2 Murashige and Skoog (MS) medium supplemented with 25 mg l^−1^ hygromycin B. Ultimately, the homozygous T3 transgenic segregants were obtained and used for subsequent component analysis. The assessment of flavonol content was referred to the methods reported previously [44].

### Gene expression analysis and correlation analysis in *M. notabilis*

Total RNA was extracted from the roots, stems, and leaves of *M. notabilis*. The method of synthesizing cDNA was mentioned above. qRT-PCR was performed on the Eppendorf realplex2 system using SYBR Green Realtime PCR Master Mix (TOYOBO, Osaka, Japan) as per the manufacturer’s protocol. The experiment was conducted through three biological replicates using beta actin gene (KJ616403.1) as an internal control. The PCR program was 95°C for 2 min, 40 cycles of 95°C for 15 s, 53°C for 15 s, and 72°C for 20 s. The relative expression levels of the genes were evaluated with the 2^-ΔΔCt^ method. The relevant primers used for qRT-PCR are listed in Table S6. Correlation analysis based on Spearman's rank method was conducted using the Metware Cloud, a free online platform for data analysis (https://cloud.metware.cn).

### Heterologous flavonoid pathway construction in *E. coli*

MnCHS was subcloned into the multiple cloning site 1 (MCS1) of pCDFDuet-1 vector between the sites of BamHI and NotI to generate the plasmid pCDFDuet-MnCHS. Mn4CL1 was then inserted into BglII and XhoI sites of pCDFDuet-MnCHS to generate plasmid pCDFDuet-MnCHS-Mn4CL1 (named p1). The plasmid p2 (pACYCDuet-MnCHR1) was constructed in the same manner. The plasmid p1 was transformed into *E. coli* BL21 to yield strain W1; p1 and p2 were cotransformed into *E. coli* to obtain strain W2. All relevant primers were presented in Table S7.

The *E. coli* strains W1 and W2 were inoculated into 3 ml of LB medium at 37°C. A portion (1%) of the preculture was inoculated into 10 ml of LB medium containing corresponding antibiotics and cultured at 37°C with shaking (110 rpm) until the OD_600_ reached 0.6. Isopropyl-β-d-thiogalactopyranoside was then added at a final concentration of 0.5 mM in order to induce the cells, followed by incubation at 16°C for 8 h. Afterward, substrates (*p*-coumaric acid and 2,4-dihydroxycinnamic acid) were added to the culture at a final concentration of 0.5 mM. After 48 h of shaking at 16°C, 0.5 ml of culture broth was harvested and extracted with 0.5 ml of ethyl acetate. The extracts were concentrated by evaporation and reconstituted in 100 μl of 80% methanol. The samples were analyzed by HPLC at a flow rate of 0.8 ml min^−1^. The mobile phases were methanol (A) and 0.1% (v/v) formic acid water (B). The gradient conditions were as follows: 0–10 min, 30%–45% A; 10–20 min, 45%–80% A; 20.1–25 min, 100% A; 25.1–30 min, 30% A. The samples were also subjected to LC–MS analysis, and the detection conditions were the same as mentioned above.

## Supplementary Material

Web_Material_uhaf171

## Data Availability

The nucleotide sequences reported in this paper have been submitted to GenBank. The accession numbers are as follows: Mn4CL1 (PV023576), Mn4CL2 (PV023577), MnCHS (PV023578), MnSTS (PV023579), MnCHR1 (PV023580), MnCHR2 (PV023581), MnCHR3 (PV023582), MnFLS (PV023583), and MnF3H (PV023584).
