# Supplementary material for: Functional characterization of key enzymes involved in the biosynthesis of distinctive flavonoids and stilbenoids in Morus notabilis
Source: Hortic Res. 2025 Jul 7;12(10):uhaf171. doi: 10.1093/hr/uhaf171 (PMC12528653; doi:10.1093/hr/uhaf171)
Supplement: Web_Material_uhaf171 [file web_material_uhaf171.zip › Supplementary-Figure.pdf]

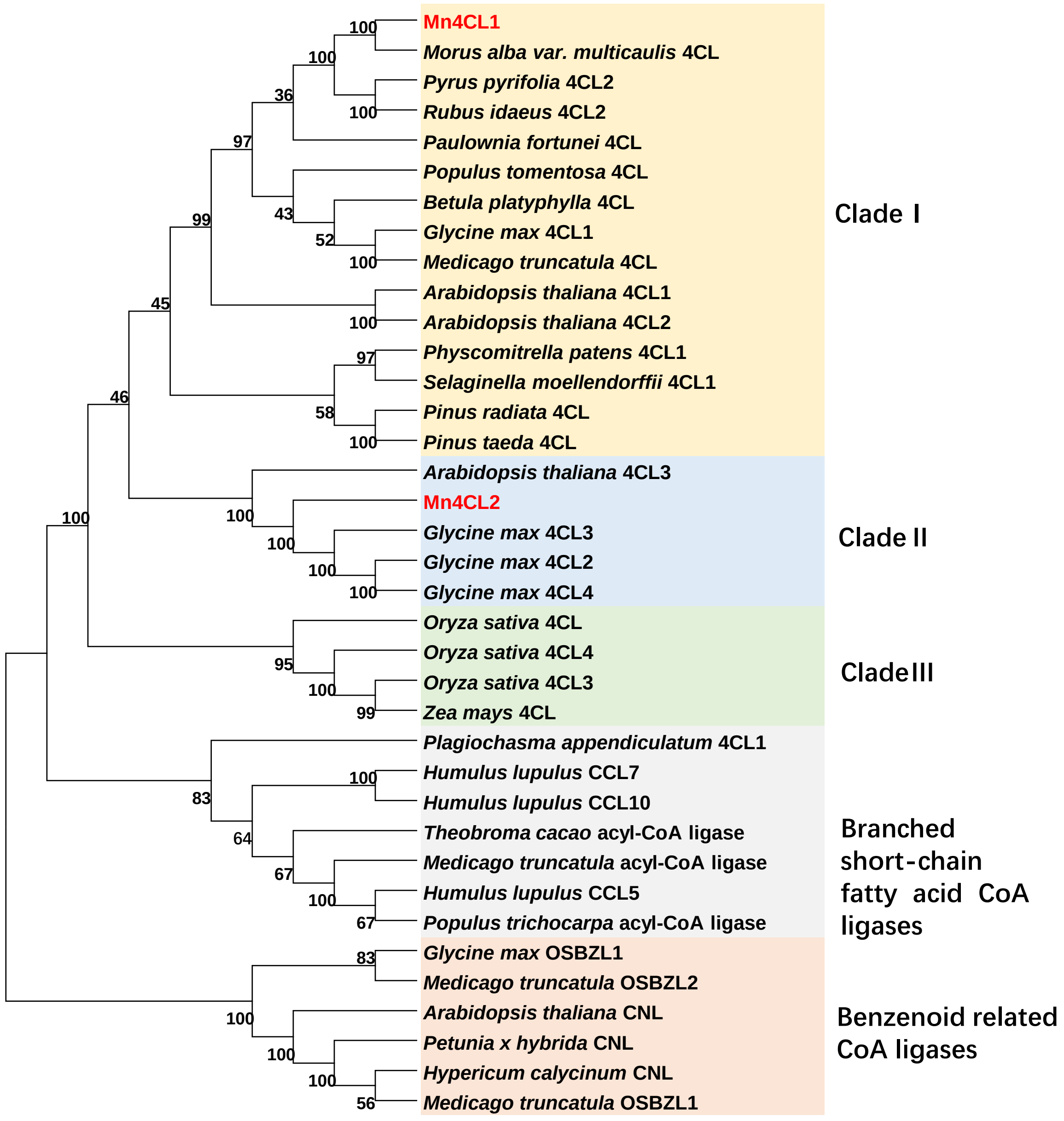

Figure S1. Phylogenetic relationship of Mn4CLs and other plant representative CoA ligases. Relevant sequence accession numbers are listed in Table S1.

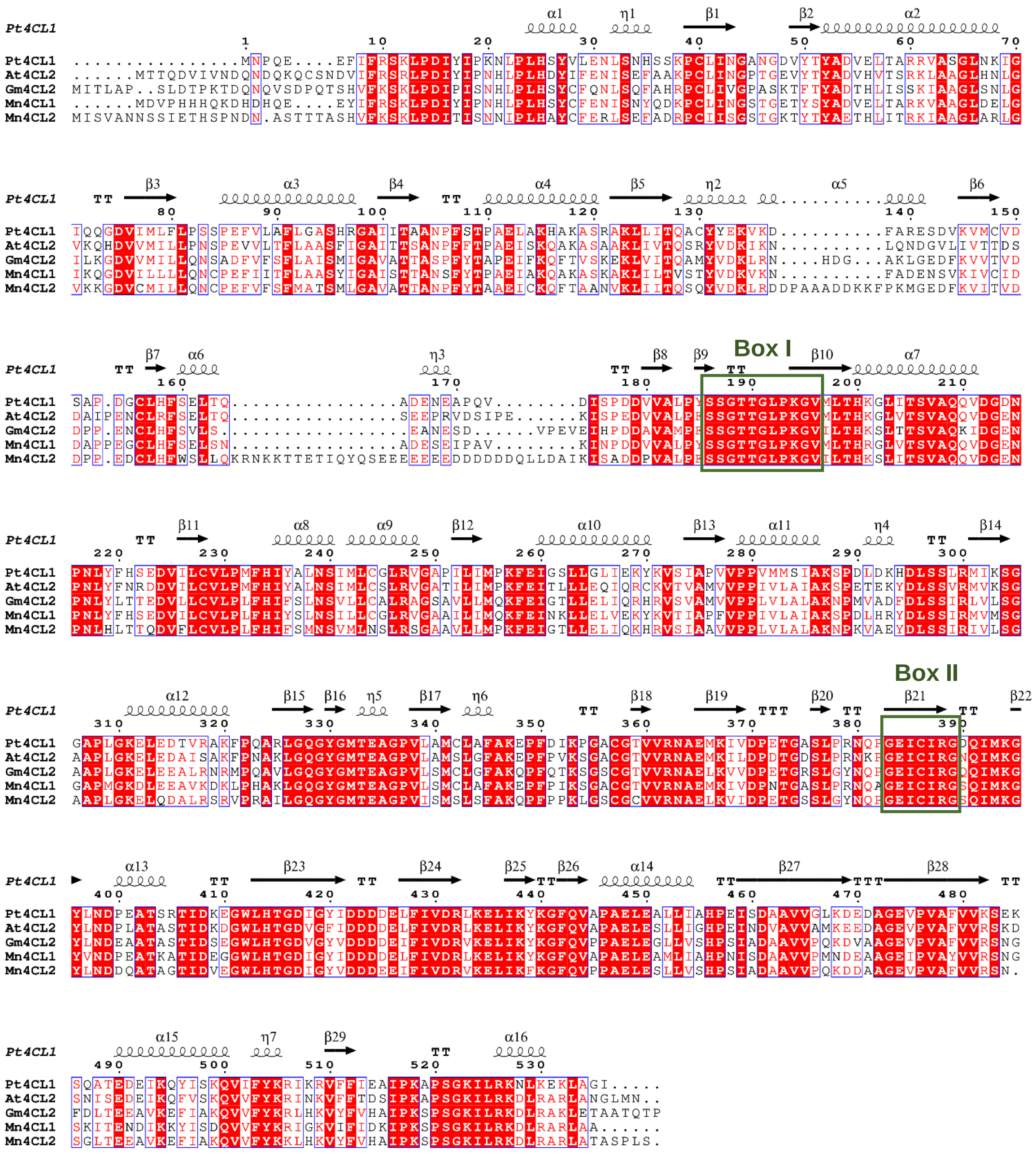

Figure S2. Sequence alignment of *M. notabilis* 4CLs with other plant 4CLs. The putative AMP-binding domain is framed in Box I and the conserved "GEICIGR" putative catalytic site is showed in Box II. The accession numbers are *Populus tomentosa* 4CL1 (AAL02145.1), *Arabidopsis thaliana* 4CL2 (NP_188761.1) and *Glycine max* 4CL2 (ACN81820.1).

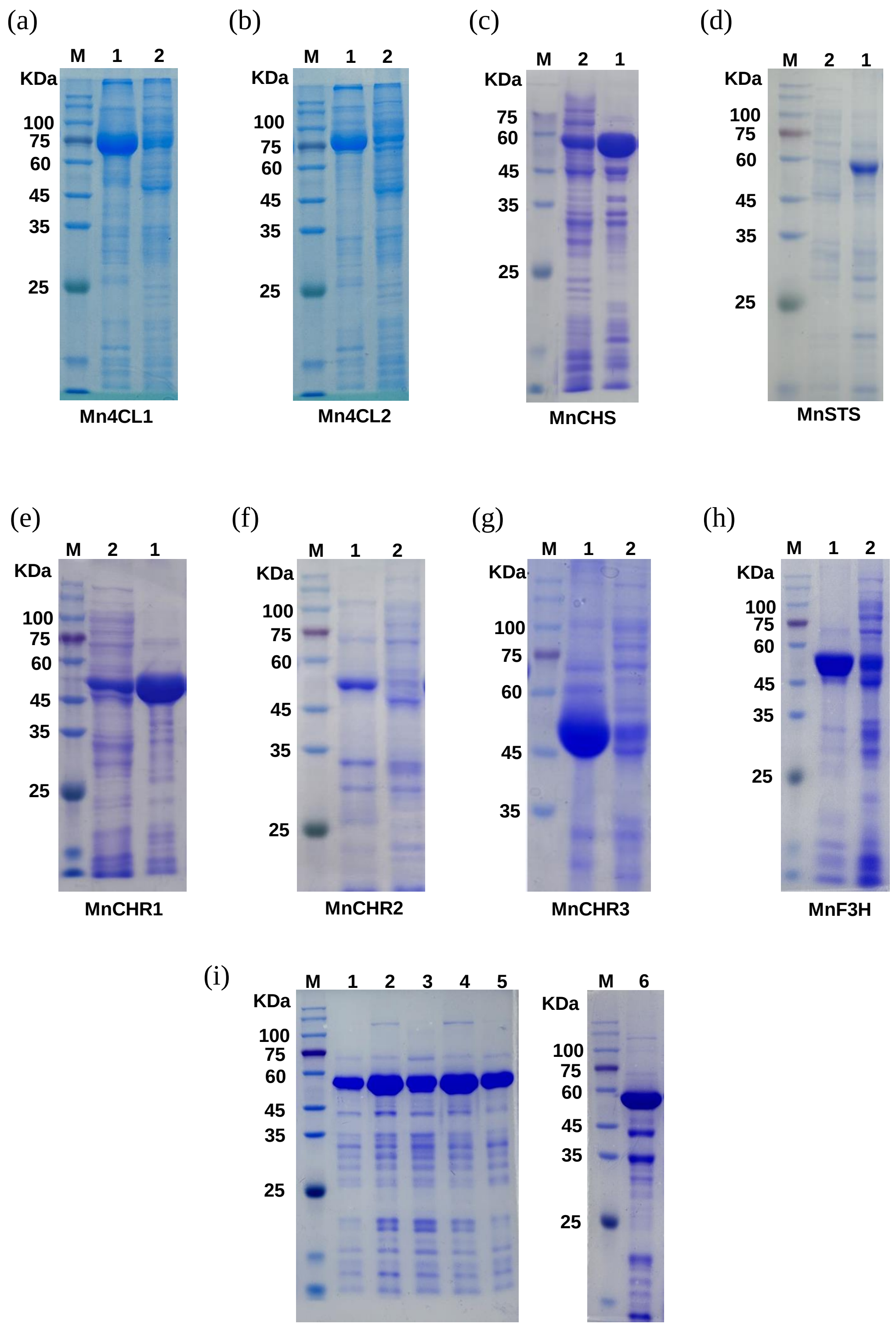

Figure S3. SDS-PAGE analysis of all recombinant proteins expressed in *E. coli* BL21. (a) - (h) M: protein marker; lane 1: purification; lane 2: supernatant. (i) M: protein marker; lane1-6: MnFLS-WT, MnFLS-T117S, MnFLS-K202V, MnFLS-L224M, MnFLS-L224P, MnFLS-T117I.

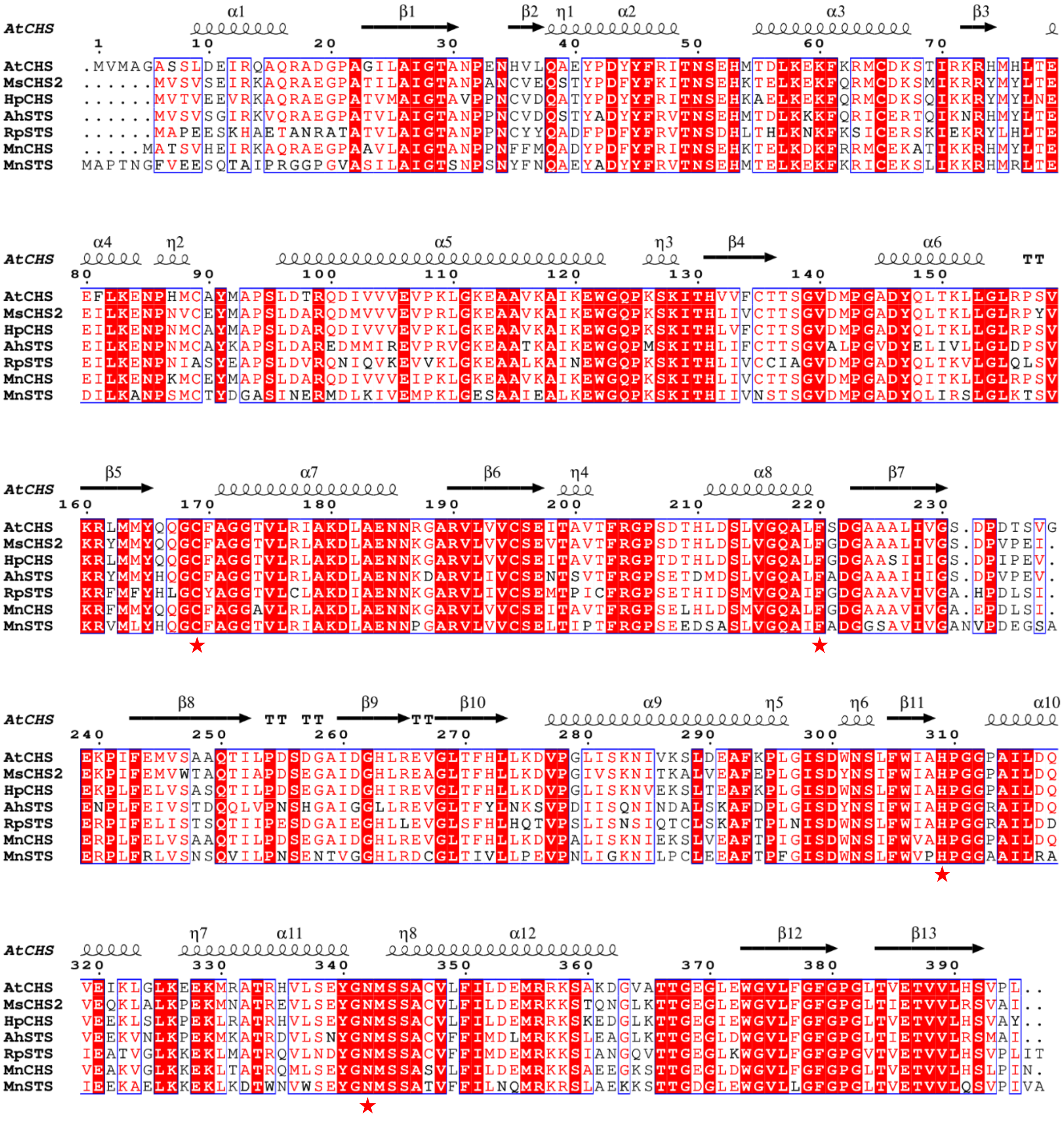

Figure S4. Sequence alignment of *M. notabilis* PKs with other plant PKs. The conserved catalytic residues in the plant type III PKS (Cys164, Phe 215, His303, and Asn336, numbering of MsCHS2) are marked with red stars. The accession numbers are *Arabidopsis thaliana* CHS (NP_196897.1), *Medicago sativa* CHS2 (P30074.1), *Hypericum perforatum* CHS (AAL67805.1), *Arachis hypogaea* STS (ADJ17764.1) and *Rheum palmatum* STS (AFX68803.1).

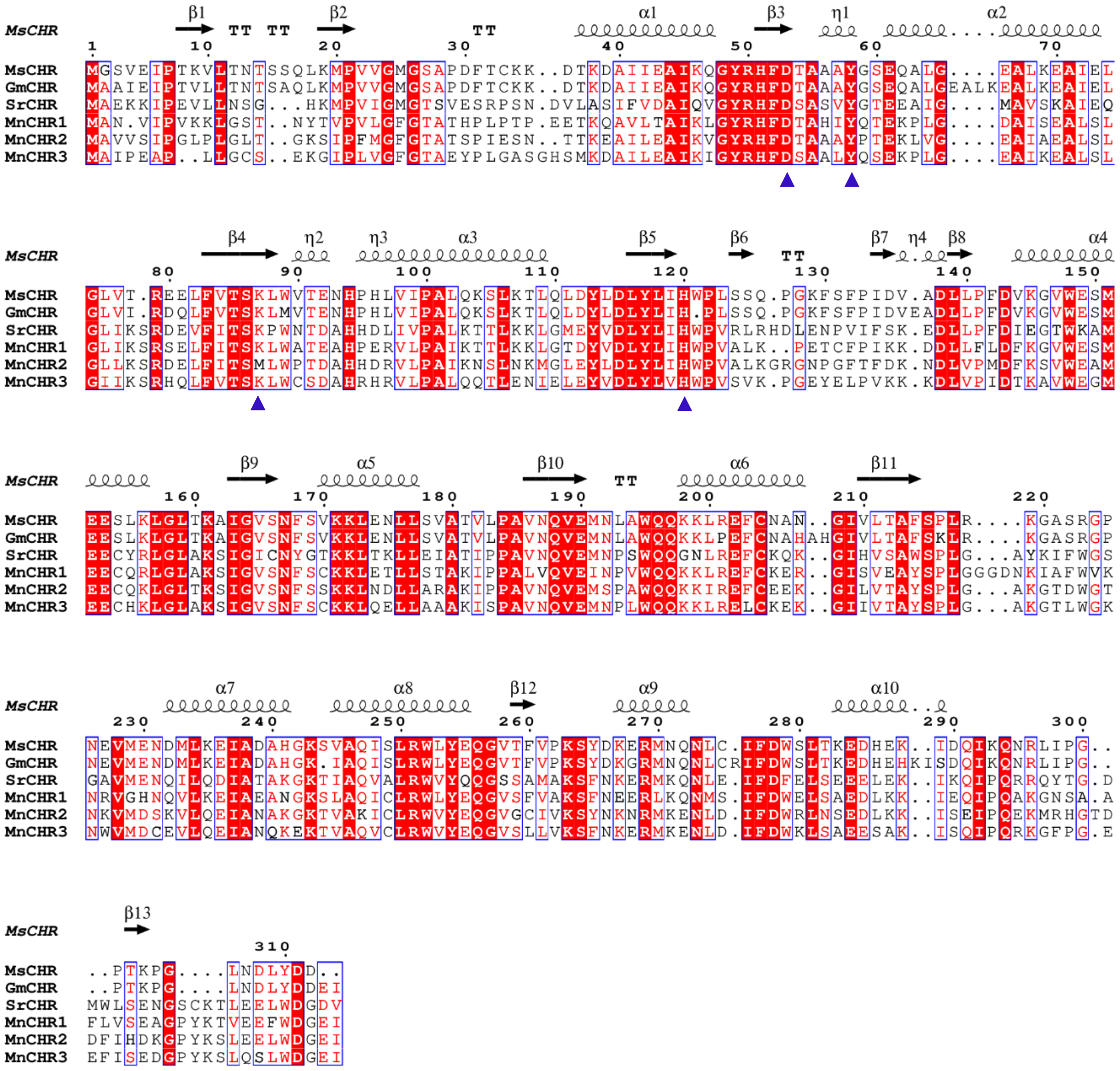

Figure S5. Sequence alignment of MnCHRs with other representative AKRs. The conserved catalytic tetrad, Asp53, Tyr58, Lys87 and His120 are marked with blue triangle. The accession numbers are *Medicago sativa* CHR (AAB41556.1), *Glycine max* CHR (AHG25321.1) and *Sesbania rostrata* CHR (CAA11226.1).

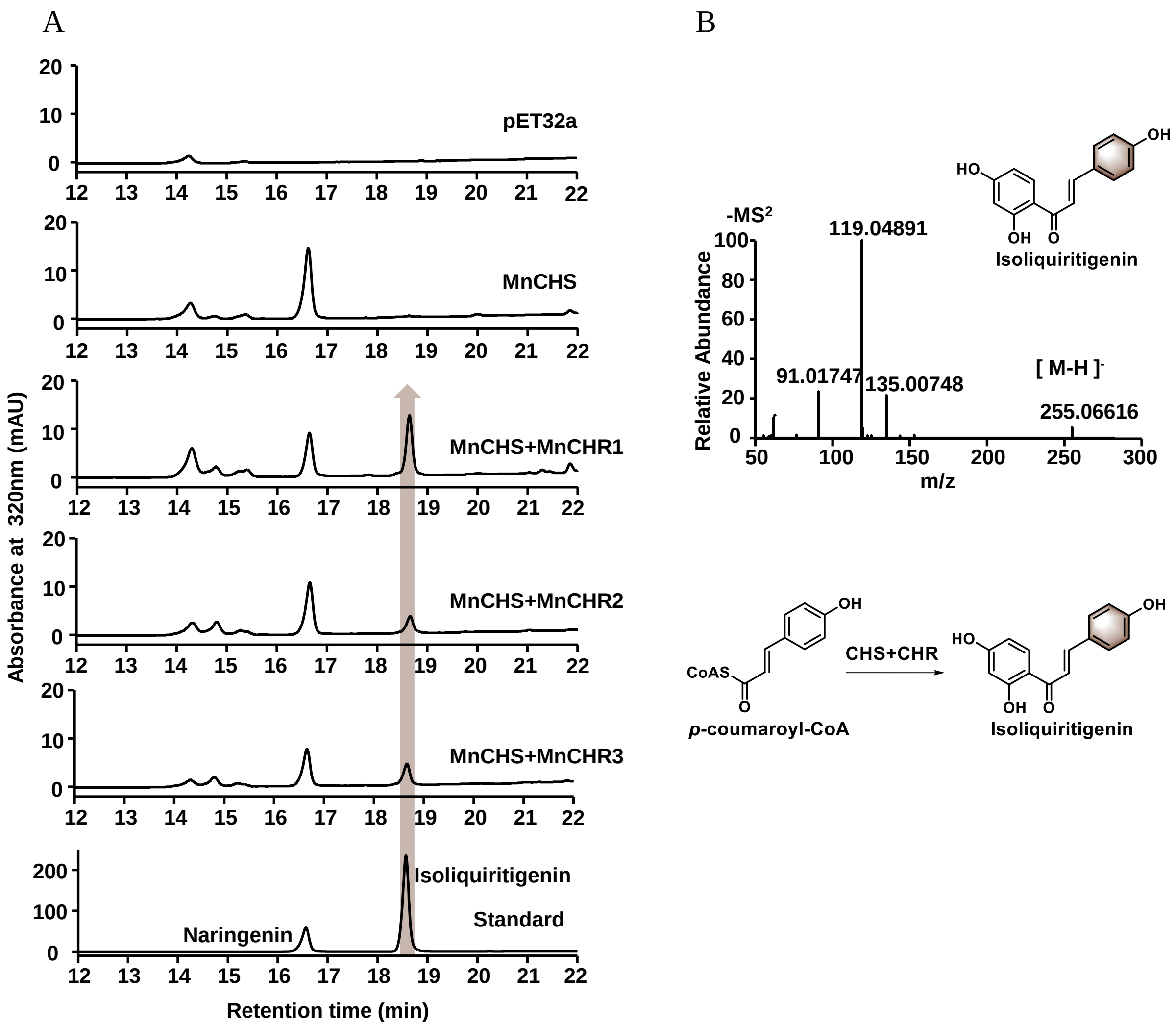

Figure S6. HPLC analysis of MnCHRs enzymatic reaction products. (A) HPLC profiles of the reaction products of using *p*-coumaroyl-CoA as substrate. (B) MS/MS fragmentation pattern of the reaction products isoliquiritigenin *in vitro* assays.

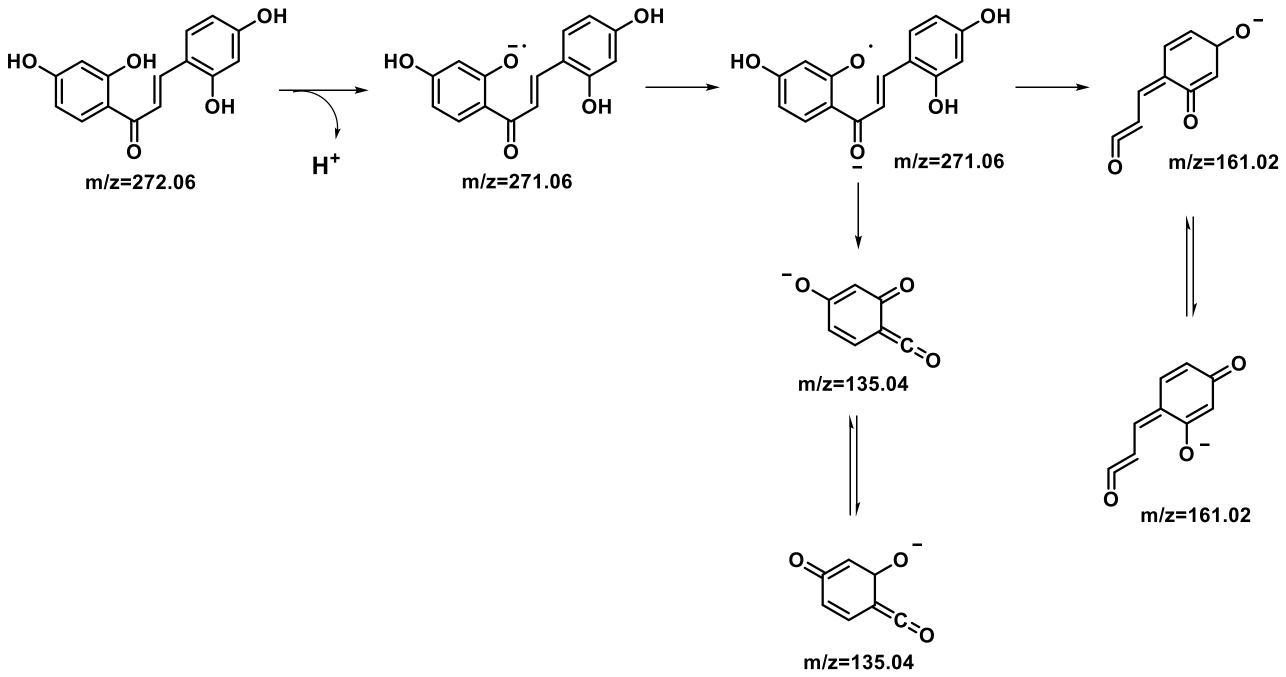

Figure S7. Proposed MS fragmentation pathway of 2,4,2',4'-tetrahydroxychalcone in negative ion mode.

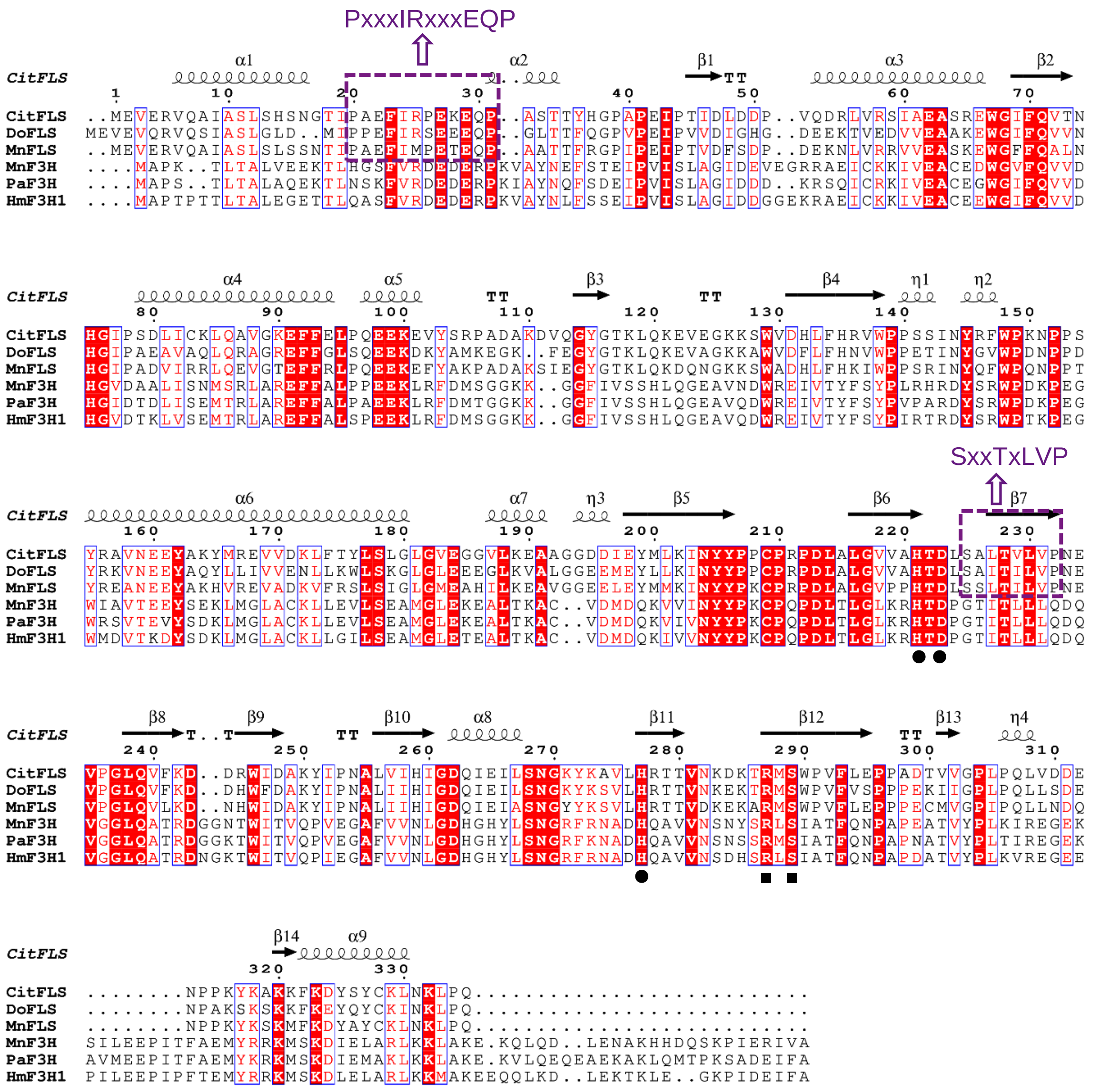

Figure S8. Sequence alignment of MnFLS, MnF3H with other functional 2-ODDs. Black dots represent ferrous iron binding residues; 2-oxoglutarate binding residues are marked with black squares. The purple dotted lines frame the motifs specific to the FLS. The accession numbers are *Dendrobium officinale* FLS (ATD53725.1), *Citrus unshiu* FLS (BAA36554.1), *Pimpinella anisum* F3H (AAX21535.1) and *Hypericum monogynum* F3H1 (WGJ63357.1).

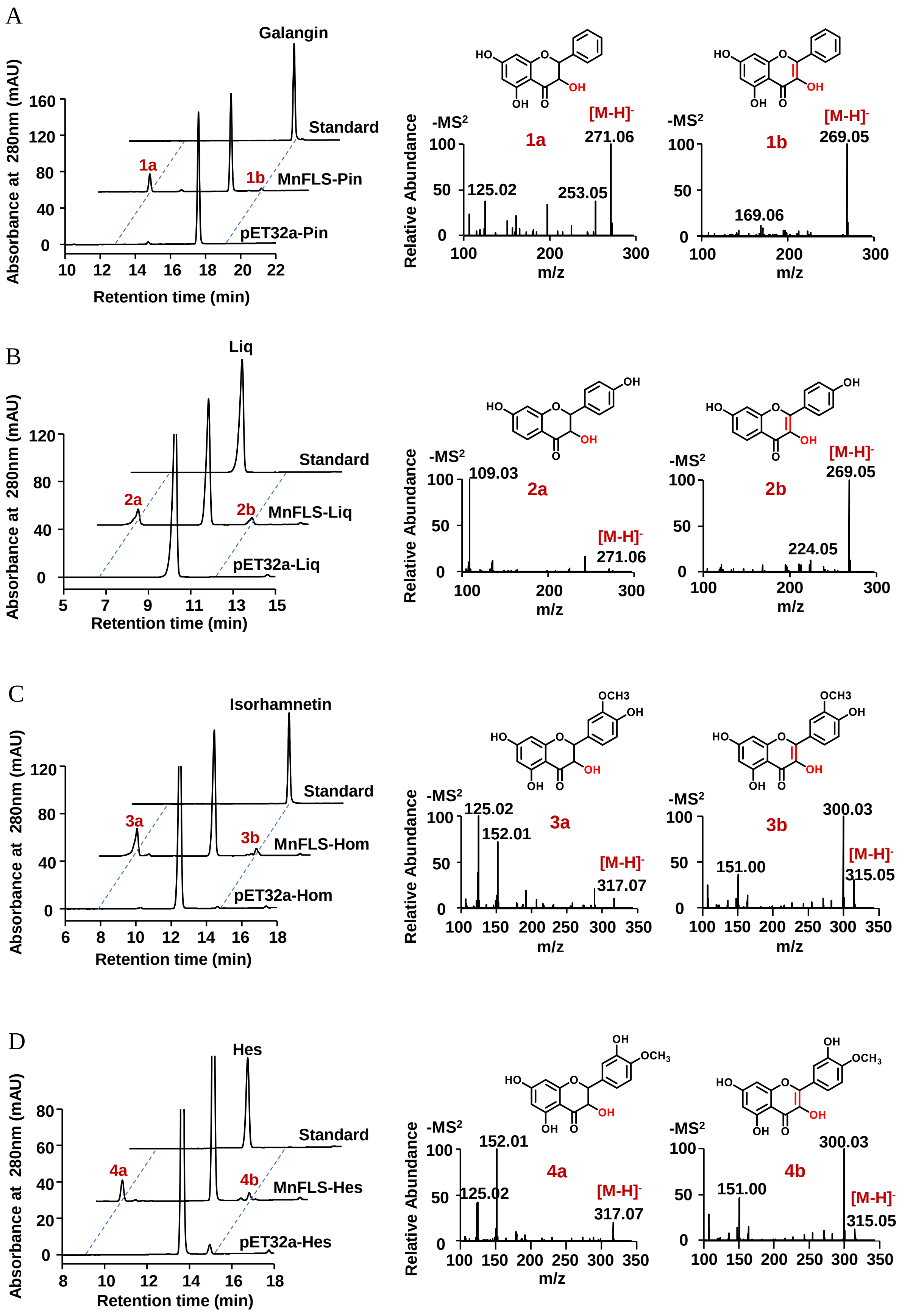

Figure S9. HPLC chromatograms of MnFLS-catalyzed reactions and MS2 spectra of the reaction products. HPLC profiles of MnFLS and typical negative ion MS2 spectra of the products using (A) Pinocembrin (Pin), (B) Liquiritigenin (Liq), (C) Homoeriodictyol (Hom) and (D) Hesperetin (Hes) as substrates. The empty vector pET32a was used as the control.

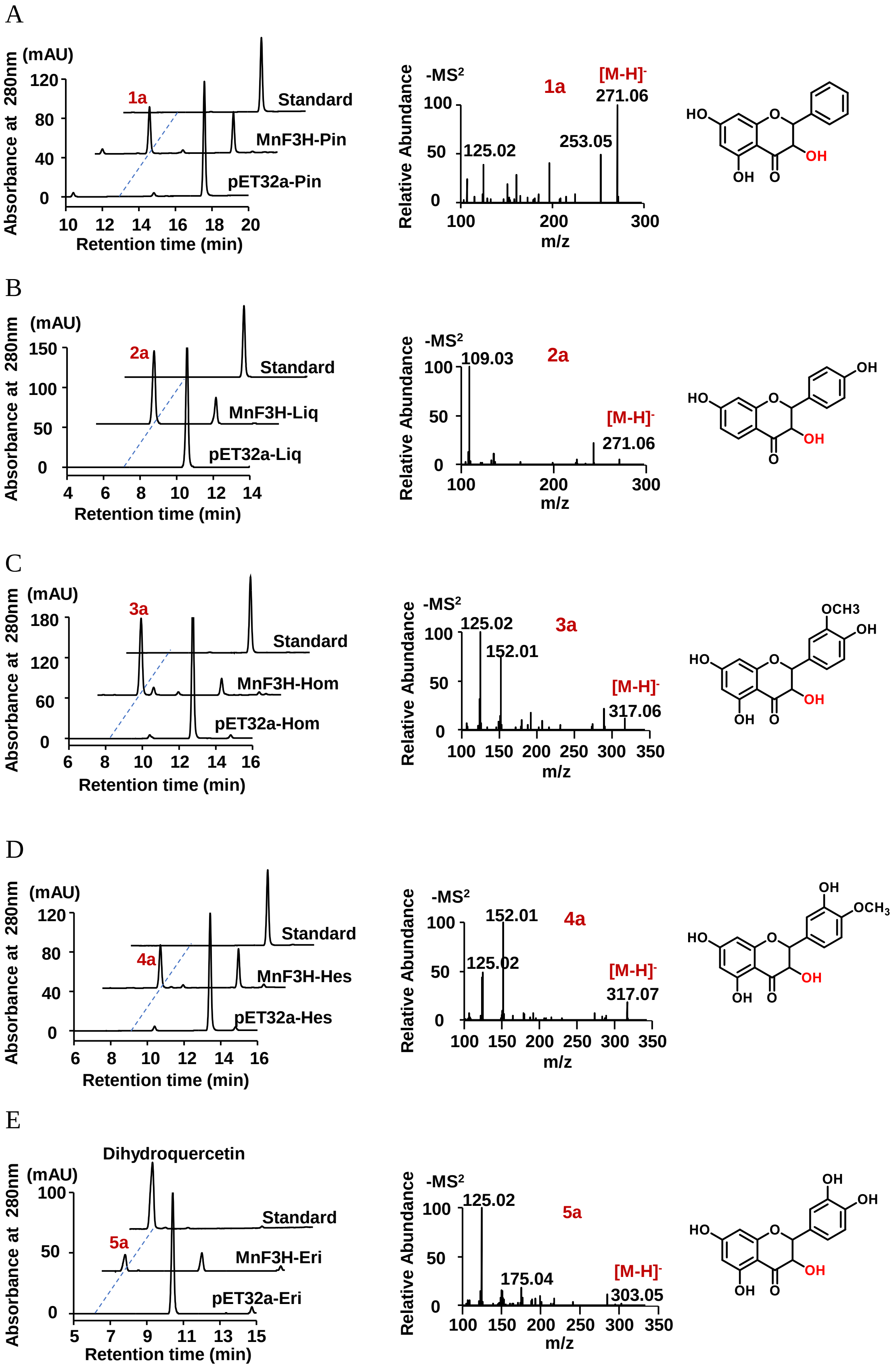

Figure S10. HPLC chromatograms of MnF3H-catalyzed reactions and MS2 spectra of the reaction products. HPLC profiles of MnF3H and typical negative ion MS2 spectra of the products using (A) Pinocembrin (Pin), (B) Liquiritigenin (Liq), (C) Homoeriodictyol (Hom), (D) Hesperetin (Hes) and (E) Eriodictyol (Eri) as substrates. The empty vector pET32a was used as the control.

A

B

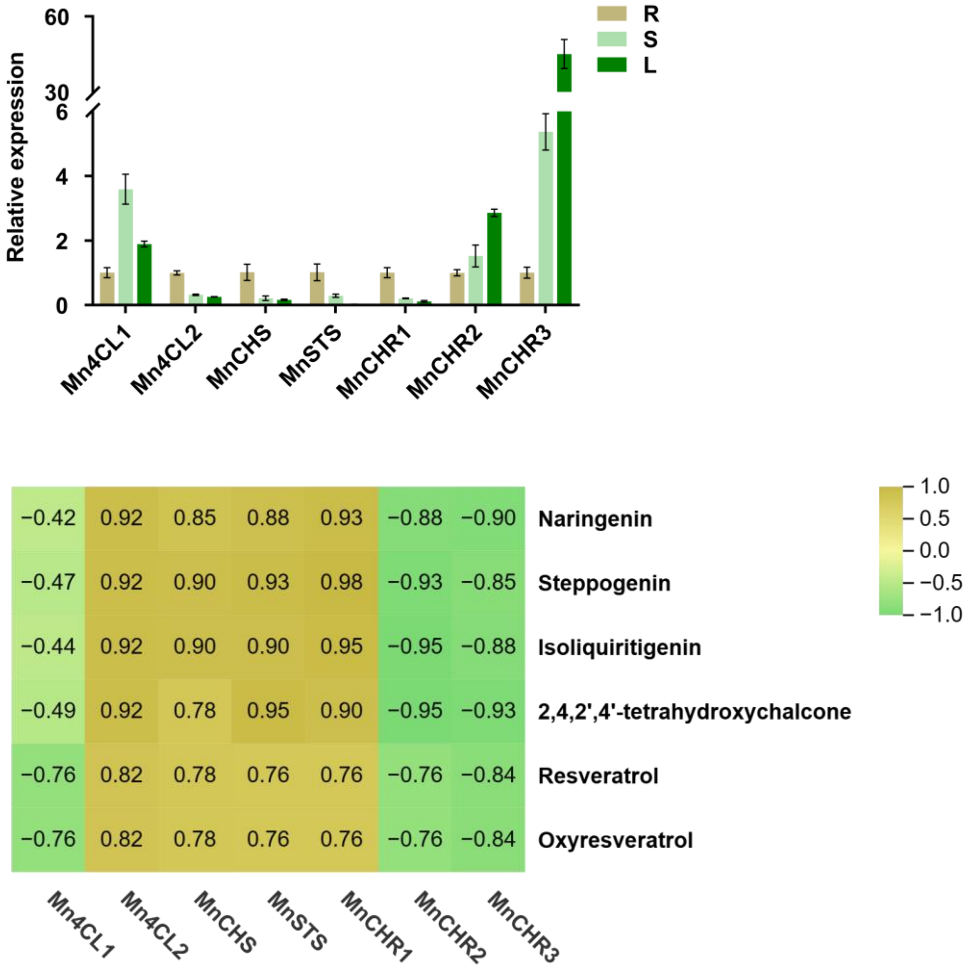

Figure S11. The relative expression analysis of key genes in plant tissues, and correlation analysis of six representative compounds with key genes expression patterns. (A) Spatial transcriptional expression profiles of key genes associated with biosynthesis of flavonoids and stilbenoids using qRT-PCR. R, root; S, stem; L, leaf. (B) The correlation analysis of six representative compounds with key genes expression patterns. The numbers in the correlation map mean the relative coefficient.

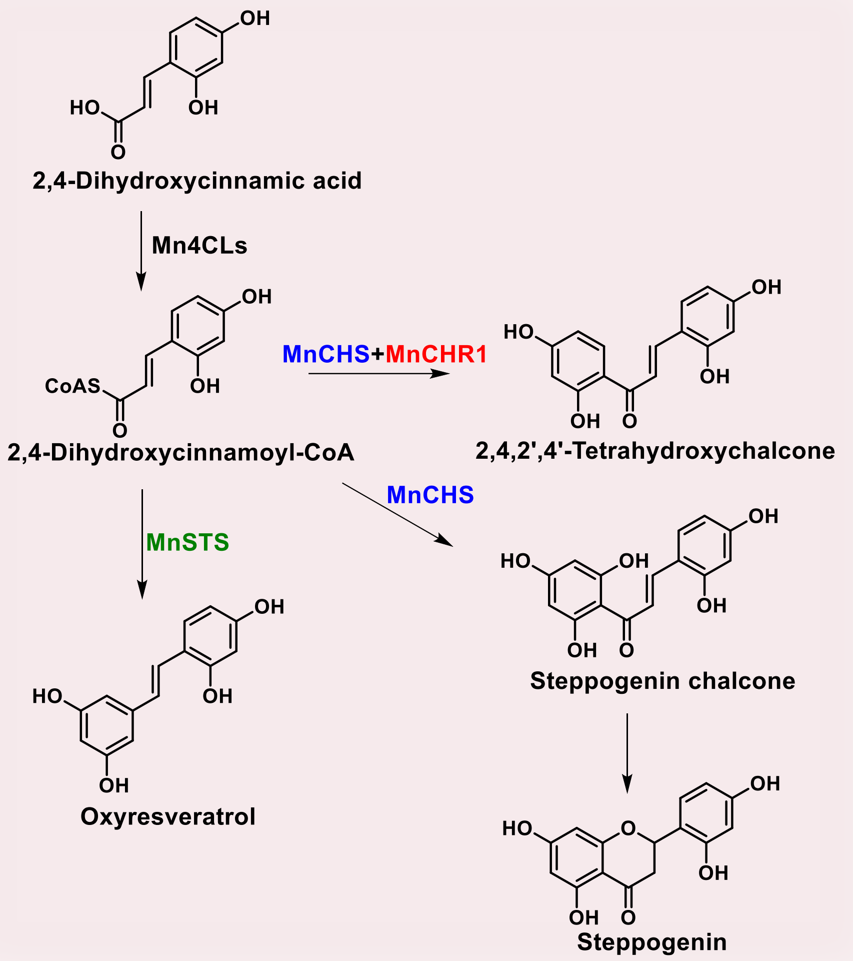

Figure S12. A possible new biosynthetic pathway for flavonoids and stilbenoids in mulberry.
