# Supplementary material for: Functional characterization of key enzymes involved in the biosynthesis of distinctive flavonoids and stilbenoids in Morus notabilis
Source: Hortic Res. 2025 Jul 7;12(10):uhaf171. doi: 10.1093/hr/uhaf171 (PMC12528653; doi:10.1093/hr/uhaf171)
Supplement: Web_Material_uhaf171 [file web_material_uhaf171.zip › Supplementary-Table.pdf]

Table S1. Sequences information used to construct the 4CL phylogenetic tree.

Gene names	Accession number of NCBI
Pyrus pyrifolia 4CL2	AFY97682.1
Rubus idaeus 4CL2	AAF91309.1
Morus alba 4CL	AHL83551.1
Paulownia fortunei 4CL	ACL31667.1
Populus tomentosa 4CL	AAL02145.1
Betula platyphylla 4CL	AAV65114.1
Glycine max 4CL1	NP_001236418
Glycine max 4CL2	ACN81820.1
Glycine max 4CL3	NP_001237270
Glycine max 4CL4	NP_001236236.2
Medicago truncatula 4CL	KEH39676.1
Arabidopsis thaliana 4CL1	NP_001077697.1
Arabidopsis thaliana 4CL2	NP_188761.1
Physcomitrella patens 4CL1	ABY21312.1
Selaginella moellendorffii 4CL1	XP_002969881.1
Pinus radiata 4CL	ACF35279.1
Pinus taeda 4CL	AAA92669.1
Arabidopsis thaliana 4CL3	NP_849844.1
Oryza sativa 4CL	CAA36850.1
Oryza sativa 4CL3	NP_001396278.1
Oryza sativa 4CL4	NP_001408603.1
Zea mays 4CL	AAS67644.1
Plagiochasma appendiculatum 4CL1	AJT43268.1
Humulus lupulus CCL7	AGA17924
Humulus lupulus CCL10	AGA17927
Theobroma cacao acyl-CoA ligase	XP_007040775.2

Medicago truncatula acyl-CoA ligase	KEH40252
Humulus lupulus CCL5	AGA17922
Populus trichocarpa acyl-CoA ligase	XP_002300662
Glycine max OSBZL1	XP_003518357.1
Medicago truncatula OSBZL1	XP_003600627.1
Medicago truncatula OSBZL2	XP_003610946.1
Arabidopsis thaliana CNL	NP_176763.1
Petunia x hybrida CNL	AEO52693.1
Hypericum calycinum CNL	AFS60176.1

Table S2. Sequences information used to construct the PKS phylogenetic tree.

Gene names	Accession number of NCBI
Humulus lupulus CHS	CAK19317.1
Koelreuteria paniculata CHS1	QXN66167.1
Actinidia chinensis CHS	AGV53049.1
Hypericum perforatum CHS	AAL67805.1
Camellia japonica CHS	BAI66465.1
Rhododendron simsii CHS	CAC88858.1
Gossypium hirsutum CHS1	NP_001314047.1
Nelumbo nucifera CHS	ADD74168.1
Vaccinium ashei CHS3	BAO58435.1
Ginkgo biloba CHS	AAS21057.1
Medicago sativa CHS2	P30074.1
Glycine max CHS	NP_001347353.1
Glycyrrhiza uralensis CHS1	ADZ45298.1
Arabidopsis thaliana CHS	NP_196897.1
Dictamnus albus CHS	CAH61575.1
Litchi chinensis CHS	ADB44077.1
Fagopyrum tataricum CHS	ACZ51475.1
Morus alba var. multicaulis CHS	AHL83549.1
Scutellaria baicalensis CHS	BAA23373.1
Zea mays CHS	CAA42763.1
Vitis pseudoreticulata STS1	ACO59912.1
Vitis vinifera STS	ABV82966.1
Vitis quinquangularis STS	ABM30193.1
Polygonum cuspidatum STS	ACC76753.1
Fallopia multiflora STS	AFP97667.1
Arachis hypogaea STS	ADJ17764.1

Rheum palmatum STS	AFX68803.1
Morus notabilis STS1	AOA48571.1
Morus notabilis STS2	AOA48572.1
Plagiochasma appendiculatum STCS1	AHY39237.1
Plagiochasma appendiculatum STCS2	AHY39238.1
Marchantia polymorpha STCS1	AAW30009.1
Marchantia paleacea STCS1	AUG98246.1
Marchantia paleacea STCS2	AUG98250.1
Marchantia paleacea CHSLK1	BAD42329.1

Table S3. Sequences information used to construct the CHR phylogenetic tree.

Gene names	Accession number of NCBI
Astragalus membranaceus CHR	ATY39973.1
Onobrychis viciifolia CHR	AEF14413.1
Medicago sativa CHR	AAB41556.1
Glycine max CHR	AHG25321.1
Lotus japonicus PKR	BAF44219.1
Glycyrrhiza echinata PKR	BAA12084.1
Glycyrrhiza glabra PKR1	BAA13113.1
Pueraria montana CHR	AAM12529.1
Vitis vinifera galacturonic acid reductase	NP_001268125.1
Fragaria x ananassa GalUR (4B4)	AAB97005.1
Papaver somniferum COR (4B3)	AAF13736.1
Zea mays DMAS	BAF03164.1
Oryza sativa DMAS (4B6)	BAF03161.1
Hordeum vulgare DMAS (4B7)	BAF03162.1
Triticum aestivum DMAS (4B8)	BAF03163.1
Sesbania rostrata CHR	CAA11226.1
Hordeum vulgare aldose reductase 4C1	P23901.1
Avena fatua aldose reductase (4C3)	Q43320
Digitalis purpurea aldose reductase (4C5)	CAC32834.1
Arabidopsis thaliana aldo-keto reductase	ABH07514.1
Arabidopsis thaliana AKR (4C9)	ABH07515.1
Malus domestica S6PDH (2A1)	P28475.1
Apium graveolens M6PR (2A2)	AAB97617.1
Oryza sativa S6PDH (2A3)	QLF98851.1
Saccharomyces cerevisiae AraDH (3C)	CAA85107.1
Pyricularia grisea aldose reductase	AAK55762.1
Yamadazyma tenuis xylose reductase	AAC25601.1
Rauvolfia serpentina perakine reductase	AAX11684.1
Zea mays putative aldo-keto reductase 2	PWZ18047.1
Dahlia pinnata AKR1-1 aldo-keto reductase	BDE26431.1
Dahlia pinnata AKR1-2 aldo-keto reductase	BDE26432.1

Table S4. Sequences information used to construct the 2-ODD phylogenetic tree.

Gene names	Accession number of NCBI
AmF3H	AAX21539.1
PaF3H	AAX21535.1
PcF3H	AAP57394.1
AtF3H	NP_190692.1
MaF3H	AOV62761.1
StF3H	AAM48289.1
MtF3H	ACR15123.1
GmF3H	NP_001236797.1
MdF3H	AAX89397.1
MsF3H	CAA55628.1
OsF3H	XP_015634635.1
ZmF3H	AAA91227.1
HmF3H1	WGJ63357.1
HmF3H2	WGJ63358.1
CsF3H	AAT68774.1
NgF3H	AFN70721.1
DcFNSI	AAX21536.1
CcFNSI	ABG78790.1
CmFNSI	AAX21538.1
AcFNSI	ABG78791.1
AgFNSI	AAX21537.1
PcFNSI	AAP57393.1
AnFNSI	DQ683352.1
GbANS	ACC66092.1
AtANS	NP_001031700.1
CrANS	XP_006283819.1
EsANS	XP_006413609.1
BrANS	XP_009136276.1
RsANS	AST09963.1

TcANS	EOY24568.1
OsANS	UUF95173
StANS	AEJ90548.1
NtANS	AWL24853.1
GmANS	NP_001240884.1
CaANS	XP_004511625.1
ZmANS	NP_001106074.1
GbFLS	ACY00393.1
CsFLS2	XP_030492734.1
CaFLS1	XP_004500586.1
CaFLS2	XP_004513519.1
OsFLS	NP_001048230.1
ZmFLS	NP_001140915.1
OcFLS1	QBQ58058.1
OcFLS2	QBQ58059.1
AcFLS	AAT68476.1
NtFLS	AFS63900.1
DoFLS	ATD53725.1
ChFLS	AIM58714.1
BnaFLS1-2	XP_013715671.1
AtFLS1	NP_001190266.1
MaFLS	QWX94156.1
CitFLS	BAA36554.1
VvFLS	BAE75806.1
MdFLS	NP_001306179.1
CmFLS	AWA82279.1
StFLS	NP_001274926.1
GtFLS	BAK09226.1
PcFLS	AAP57395.1

Table S5. Primers used for the genes cloning in this study.

**Primer name**	**Primer sequences (5' to 3')**
Mn4CL1-*Bgl* II-F	GAAGATCTGATGGACGTCCCCCACCACCA
Mn4CL1-*Xho* Ⅰ-R	CCGCTCGAGTTATGCAGCAAGTCTTGCTC
Mn4CL2-*Hin*d III-F	CCCAAGCTTGCATGATTTCCGTAGCTAACAA
Mn4CL2-*Xho* Ⅰ-R	CCGCTCGAG TTAGCTCAAAGGGGAAGCTG
MnCHS-*Bam*H Ⅰ-F	CGGGATCCATGGCGACCTCCGTCCACGA
MnCHS-*Xho* Ⅰ-R	CCGCTCGAGTTAATTAATGGGAAGGCTGT
MnSTS-*Bam*H Ⅰ-F	CGGGATCCATGGCGCCGACTAACGGGTT
MnSTS-*Xho* Ⅰ-R	CCGCTCGAGCTAGGCAACAATAGGGACAC
MnCHR1-*Bam*H Ⅰ-F	CGGGATCCATGGCCAACGTTATTCCAGT
MnCHR1-*Xho* Ⅰ-R	CCGCTCGAGTTAAATCTCTCCATCCCAGA
MnCHR2-*Bam*H Ⅰ-F	CGGGATCCATGGCAGTAGTAAGCATCCC
MnCHR2-*Sal* I-R	ACGCGTCGACTTAAATCTCTCCATCCCACA
MnCHR3-*Bam*H Ⅰ-F	CGGGATCCATGGCAATTCCTGAAGCCCC
MnCHR3-*Sal* I-R	ACGCGTCGACTCAGATCTCGCCATCCCAAA
MnFLS-*Bam*H Ⅰ-F	CGGGATCCATGGAGGTTGAGA GAGTTCA
MnFLS-*Xho* Ⅰ-R	CCGCTCGAGTCACTGGGGAAGCTTGTTGA
MnF3H-*Bam*H Ⅰ-F	CGGGATCC ATGGCTCCCAAAACTCTCAC
MnF3H-*Xho* Ⅰ-R	CCGCTCGAGTTAAGCAACAATCCGCTCAA

Table S6. The relevant primers used for qRT-PCR in this study.

Primer name	Primer sequences (5' to 3')
Mn4CL1-RT-F	TGAGGAGGCCGTCAAGGATA
Mn4CL1-RT-R	CTTGGAAGCGATGCTCCAGT
Mn4CL2-RT-F	TCCATTGAAACCCACTCTCCC
Mn4CL2-RT-R	AAGTGTAGGTCTTGCCGGTG
MnCHS-RT-F	GCCGCTTTTTGAGCTGGTTT
MnCHS-RT-R	TGCCTCCACCAGACTCTTCT
MnSTS-RT-F	TGCCATTTTGAGGGCGATTG
MnSTS-RT-R	CAACAATAGGGACGCTCTGC
MnCHR1-RT-F	TCGGACACAATCAGGTGCTC
MnCHR1-RT-R	ACAGTCTTGTAAGGGCCTGC
MnCHR2-RT-F	GGCTGTCAACCAAGTGGAGA
MnCHR2-RT-R	TGCTCATACACCCATCGCAA
MnCHR3-RT-F	AGTGTGGGAAGGCATGGAAG
MnCHR3-RT-R	AGCACCTCGCAGTCCATTAC
Mn-Actin-F	ATTCCGATGTCCTGAAGTCCT
Mn-Actin-R	GCTGTGATCTCCTTGCTCATC

Table S7. The primers used for strains construction and heterologous expression.

**Primer name**	**Used for**	**Primer sequences (5' to 3')**
Mn4CL1-CD-*Bgl* II-F	Strain Construction	GAAGATCTCATGGACGTCCCCCACCACCA
Mn4CL1-CD-*Xho* Ⅰ-R	Strain Construction	CCGCTCGAGTTATGCAGCAAGTCTTGCTC
MnCHS-CD-*Hin*d III-F	Strain Construction	CCCAAGCTTATGGCGACCTCCGTCCACGA
MnCHS-CD-*Not* I-R	Strain Construction	ATAAGAATGCGGCCGCTTAATTAATGGGAAGGCT GT
MnCHR-CYC-*Bgl* II-F	Strain Construction	GAAGATCTC ATGGCCAACGTTATTCCAGT
MnCHR-CYC-*Xho* Ⅰ-R	Strain Construction	CCGCTCGAGTTAAATCTCTCCATCCCAGA
attB-MnFLS-F	Gateway	GGGGACAAGTTTGTACAAAAAAGCAGGCTTAACC ATGGAGGTTGAGAGAGTTCA
attB-MnFLS-R	Gateway	GGGGACCACTTTGTACAAGAAAGCTGGGTC TCACTGGGGAAGCTTGTTGA

Table S8. The primers used for the site-directed mutagenesis vector construction.

Primer name	Primer sequences (5' to 3')
MnFLS-T117S-F	CATTGAAGGGTACGGGAGCAAGCTCCAAAAGGACC
MnFLS-T117S-R	GGTCCTTTTGGAGCTTGCTCCCGTACCCTTCAATG
MnFLS-T117I-F	GAAGGGTACGGGATTAAGCTCCAAAAG
MnFLS-T117I-R	CTTTTGGAGCTTAATCCCGTACCCTTC
MnFLS-K202V-F	GGAGTTAGAGTACATGATGGTGATCAATTACTATCCGAAG
MnFLS-K202V-R	CTTCGGATAGTAATTGATCACCATCATGTACTCTAACTCC
MnFLS-L224M-F	CCGCACACTGACATGTCGTCGCTAAC
MnFLS-L224M-R	GTTAGCGACGACATGTCAGTGTGCGG
MnFLS-L224P-F	GTTCCGCCGCACACTGACCCGTCGTCGCTAACAATTTTG
MnFLS-L224P-R	CAAAATTGTTAGCGACGACGGGTCAGTGTGCGGCGGAAC
